# Going Asexual: A Survey of Mites of the Genus *Thyreophagus* (Acari: Acaridae) Revealing a Large Number of New Parthenogenetic Species in the Holarctic Region

**DOI:** 10.3390/life13112168

**Published:** 2023-11-05

**Authors:** Pavel B. Klimov, Vasiliy B. Kolesnikov, Emilie P. Demard, Clive S. A. Stinson, Jonas Merckx, Marcus V. A. Duarte, Luiz Gustavo A. Pedroso, Alexander A. Khaustov, James Leslie Myers-Hansen, Felix L. Wäkers, Dominiek Vangansbeke

**Affiliations:** 1 Lilly Hall of Life Sciences, Purdue University, G-225, 915 W State St, West Lafayette, IN 47907, USA; jmyersha@purdue.edu; 2 X-Bio Institute, Tyumen State University, 25 Lenina St. Str., 625003 Tyumen, Russia; jukoman@yandex.ru (V.B.K.);; 3 All-Russian Research Institute of Plant Protection, 396030 Voronezh, Russia; 4 Citrus Research and Education Center, University of Florida, 700 Experiment Road Station, Lake Alfred, FL 33850, USA; 5 Beneficial Insectary Inc., Redding, CA 96003, USA; clivebugsme@outlook.com; 6 Biobest Sustainable Crop Management, R&D, 2260 Westerlo, Belgium; jonas.merckx@biobestgroup.com (J.M.);; 7 Biodiversity Inventory for Conservation NPO (BINCO), Walmersumstraat 44, 3380 Glabbeek, Belgium; 8 Departamento de Zoologia, Universidade Estadual Paulista, Av. 24-A, 1515, Rio Claro 13506-900, SP, Brazil; luizgustavopedroso@gmail.com

**Keywords:** astigmatid mites, parthenogenetic species, new species, morphology, systematics, North America, Europe, factitious food source

## Abstract

Mites of the genus *Thyreophagus* (Acari: Acaridae) are distributed worldwide; they inhabit concealed habitats and include several beneficial and economically important species. However, species identification is difficult because many species are poorly described or delimited and their phoretic stages are unknown or uncorrelated. Furthermore, *Thyreophagus* is interesting because it includes entirely asexual (parthenogenetic) species. However, among the 34 described species of *Thyreophagus*, the asexual status is confirmed through laboratory rearing for only two species. Here, we provide detailed descriptions of five new species from North America (four) and Europe (one) based on adults and phoretic heteromorphic deutonymphs. Four of these species were asexual, while one was sexual. For most of these mites, the asexual status was confirmed and phoretic deutonymphs were obtained through rearing in the lab. We show that asexual mites retain seemingly functional copulatory and sperm storage systems, indicating that these lineages have relatively short evolutionary lifespans. One North American species, *Thyreophagus ojibwe*, was found in association with the native American chestnut *Castanea dentata*, suggesting a possibility that this mite can be used to control chestnut blight in North America. We also provide a diagnostic key to females, males, and heteromorphic deutonymphs of the *Thyreophagus* species in the world.

## 1. Introduction

The genus *Thyreophagus* (Rondani, 1874) is distributed worldwide, except in Antarctica [1,2,3,4,5]. There are 39 nominal species and one subspecies inhabiting various environments; however, most commonly, they are found in subcortical habitats and stored food [1,2,6,7,8,9]. Some species of the genus are beneficial and used as a factitious food source for the mass rearing of predacious phytoseiid mites used for biological control [10,11,12]. The Palearctic species, *Thyreophagus corticalis* (Michael, 1885) is beneficial because it is a natural vector of a hypovirus pathogenic to the ascomycete fungus *Cryphonectria parasitica* that causes chestnut blight, a dangerous disease of chestnuts in temperate regions in the northern hemisphere [13]. The hypovirus infects *C. parasitica* and reduces its parasitic growth, leading to the spontaneous recovery of infected chestnut trees, *Castanea sativa*, from the disease in Europe [14,15]. When *C. parasitica* was inadvertently introduced to North America, it caused the functional extinction of the American chestnut *Castanea dentata* [16]. Efforts to find remedies to alleviate the destructive attacks of this pathogen have been ongoing for many decades, but no practical and widely successful solution had been discovered to date [17].

Since most species of *Thyreophagus* live in concealed habitats, they have been generally overlooked and understudied by researchers in comparison to most other acarid mite genera. The lack of correlation of adult and deutonymphal stages for many species; the presence of old, insufficiently described, unrecognizable taxa; the lack of types; and unclear species boundaries are cited as being the major impediments to the systematics of *Thyreophagus* [8]. As an example, only four species are recognizable in both adult and deutonymphal stages: *Th. Australis* (Clark, 2009), *Th. Corticalis*, *Th. Entomophagus*, and *Th. Calusorum* Klimov, Demard, Stinson, Duarte, Wäckers, and Vangansbeke, 2022 [8,18,19,20]. One remarkable feature of the genus *Thyreophagus* is the presence of both sexual and asexual species, although only a few species from the latter group are known to date: *Th. Calusorum*, *Th. Plocepasseri* (Klimov, Mwangi, Vangansbeke, 2020), and an unnamed species from a wasp nest in Japan [9]. Asexual species have been reared in cultures for many generations with no males detected.

Here, we provide detailed descriptions of five new species from North America (four) and Europe (one) based on adult and phoretic heteromorphic deutonymphs. Four of these species were asexual, while one was sexual. The asexual status for most of these mites was confirmed through lab rearing. One North American species was found in association with the native American chestnut *Castanea dentata*, thus opening avenues for further research investigating whether the mite also can vector a hypoviruses that can infect and control the fungal pathogen of the tree. We demonstrate that females of asexual species retain functional structures responsible for insemination and sperm storage, with no signs of morphological reductions observed in the spermatheca. This suggests that these taxa are recent and probably evolutionary short-lived asexuals, thus meriting further genomic research into the potential causes of their asexual mode of reproduction. We also provide a diagnostic key to females, males, and heteromorphic deutonymphs for all known *Thyreophagus* species in the world.

## 2. Materials and Methods

Fallen tree branches were collected from forest litter and processed in the lab. Feeding stages of live mites were sampled in subcortical spaces under a Zeiss Stemi DV4 dissecting microscope. Voucher specimens were mounted on slides, preserved in ethanol, or cultured (room temperature, RH 85%) using bran as a food source, with the addition of baker’s yeast (80% bran, 20% yeast by volume). The culture was harvested and subcultured several times to obtain a large number of specimens and heteromorphic deutonymphs. The specimens were preserved in ethanol, cleared in Nesbitt’s fluid for 1–2 days, mounted in Hoyer’s medium, and dried at 60 °C for 7 days [21]. To control for potential environmentally induced variations due to culturing [22], both original specimens collected in the wild and cultured specimens were examined (see details below).

Images were taken using a Nikon Eclipse E800 microscope equipped with a DIC optic and a Tucsen Discovery CH30 digital camera, and a Zeiss Axio Imager.A2 equipped with a DIC optic and Axiocam 305 color camera. Images were taken from multiple focal planes and assembled in Helicon Focus 7.6.4 Pro (algorithm B, rarely A) with subsequent manual editing to add missing fine detail from the individual focal planes (retouching). Parts of the layered images were combined in Adobe Photoshop 22.2.0. Line drawings were created in Photoshop using microphotographs as the background.

The idiosomal chaetotaxy followed Griffiths et al. [23]; the terminology of coxisternal setae followed Norton [24]; for appendages, the chaetotaxy and solenidiotaxy followed Grandjean for palps [25] and legs [26]. Designations of tarsal dorsoapical setae on legs III–IV followed Klimov et al. [7]. All measurements are presented in micrometers (μm).

## 3. Descriptions of New Species

*Thyreophagus ais* Klimov, Kolesnikov, Demard, Vangansbeke sp. n.

(Figure 1, Figure 2, Figure 3, Figure 4, Figure 5, Figure 6, Figure 7 and Figure 8).

urn:lsid:zoobank.org:pub:7C7FEAA7-EB57-4A12-A489-196D2BEEA5D9.

**Type material**. Holotype: One female—lab culture on bran, harvested 26 February 2021, culture started from specimens originated from the USA, Florida, Fort Pierce, branches on ground in a small wooded area, subcortical, 27°25′34.5″ N 80°24′22.7″ W, 20 November 2020, Emilie Demard coll., BMOC 20-0101-014#S1.1. Paratypes: 3f (S1.2,3; S3.1), 6 HDNs (S4.1; S5.1; S6.1-4)—same data; 2f—original wild sample (see above).

**Depository**. Holotype, paratypes—University of Michigan, Museum of Zoology, Ann Arbor, Michigan, USA.

**Etymology**. The new species is named after the Ais people who lived in the eastern coastal area of central Florida (including the type locality of the new species) but went extinct after the arrival of European colonizers [27].

**Habitat***. Thyreophagus ais* lives under the bark of small fallen branches of deciduous trees in wooded areas. These branches are typically in the initial stages of decomposition, with approximately 80% of their natural sapwood retaining a white color, and 20% displaying brown discoloration, indicating the ongoing decomposition process. Additionally, the bark usually exhibits boreholes from wood-boring beetles.

**Description**

**Figure 1:**
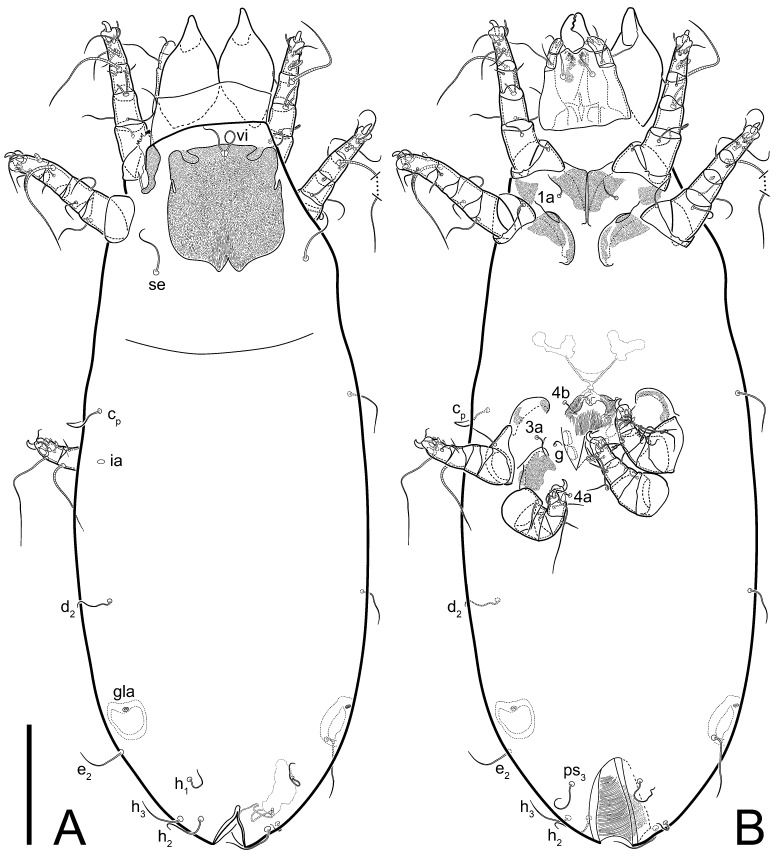
*Thyreophagus ais* sp. n., female (holotype): (**A**)—dorsal view; (**B**)—ventral view. Scale bar: 100 μm.

**Figure 2:**
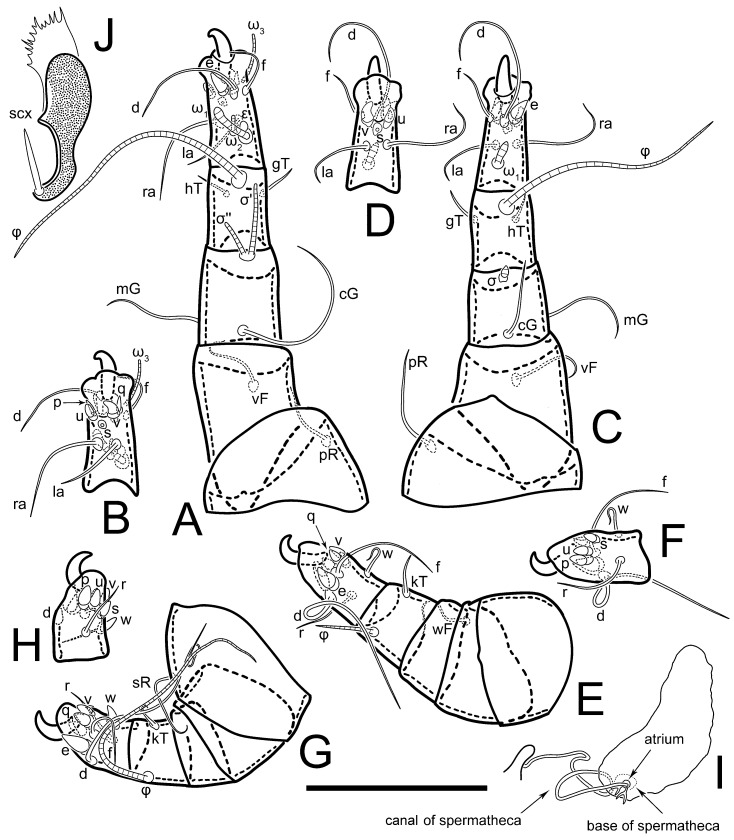
*Thyreophagus ais* sp. n., female (holotype): A—leg I, dorsal view; B—tarsus I, ventral view; C—leg II, dorsal view; D—tarsus II, ventral view; E—leg III, dorsolateral view; F—tarsus III, ventrolateral view; G—leg IV, dorsolateral view; H—tarsus IV, ventrolateral view; I—spermatheca; J—supracoxal sclerite and Grandjean’s organ. Scale bar: 50 μm.

**Figure 3:**
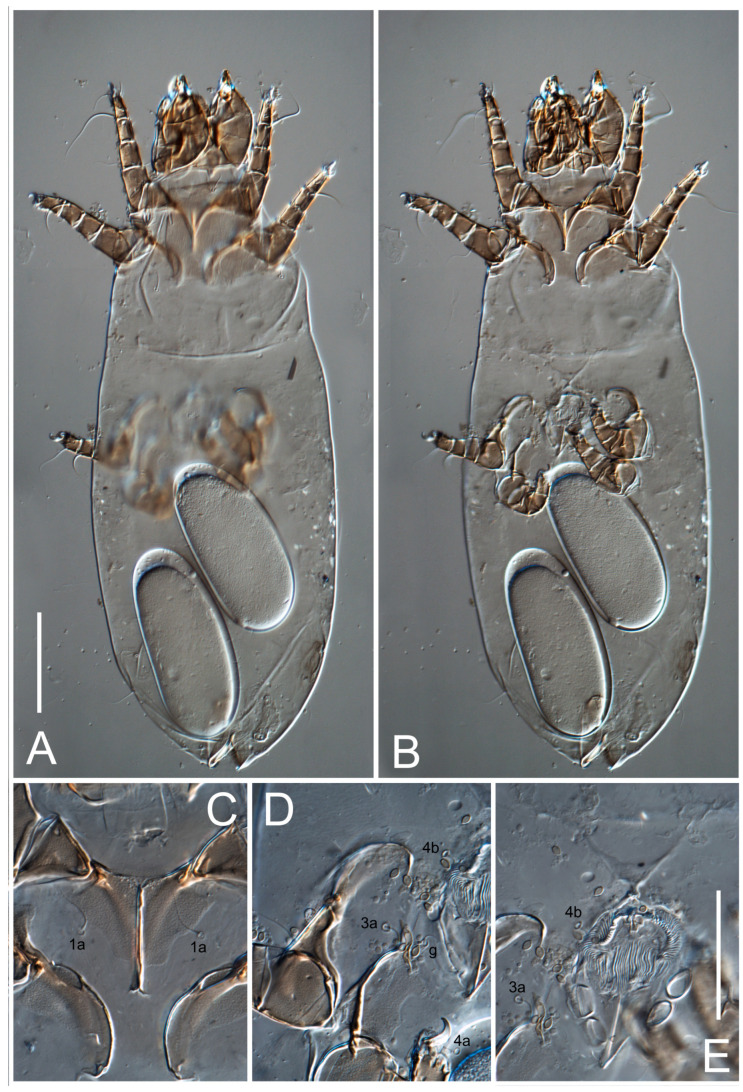
*Thyreophagus ais* sp. n., female (holotype), DIC images: (**A**)—dorsal view; (**B**)—ventral view; (**C**)—coxal fields I–II; (**D**)—coxal fields III–IV; (**E**)—ovipore and coxal fields III–IV. Scale bar: 50 μm.

**Figure 4:**
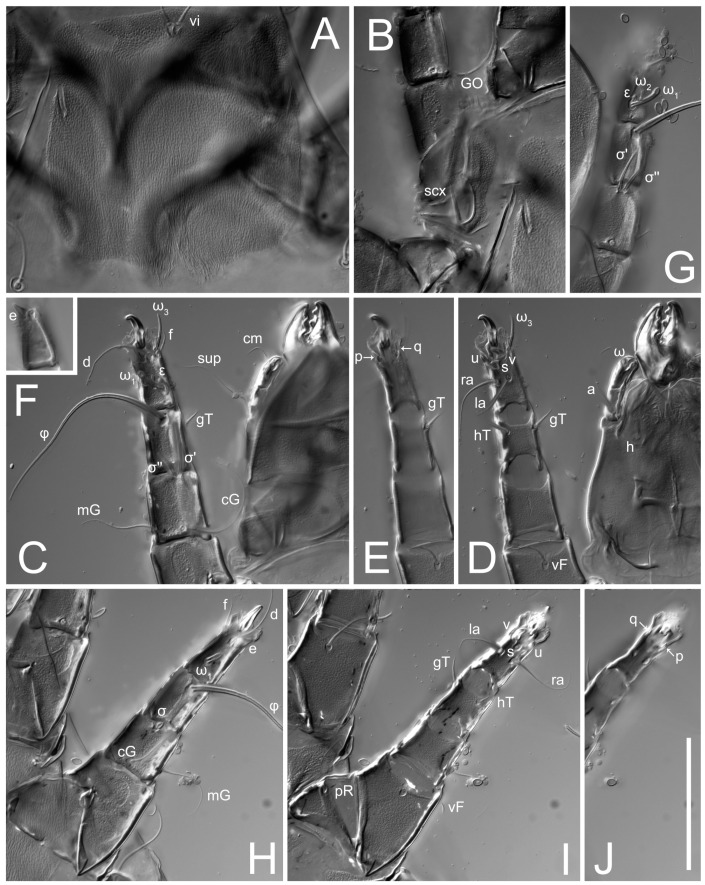
*Thyreophagus ais* sp. n., female (holotype), DIC images: (**A**)—prodorsal shield; (**B**)—supracoxal sclerite and Grandjean’s organ; (**C**)—leg I and gnathosoma (part), dorsal view; (**D**)—leg I and gnathosoma (part), ventral view; (**E**)—leg I, ventral view; (**F**)—tarsus I, dorsal view; (**G**)—leg I, dorsal view; (**H**)—leg II, dorsal view; (**I**,**J**)—leg II, ventral view; tarsus II, ventral view. Scale bar: 50 μm.

**Figure 5:**
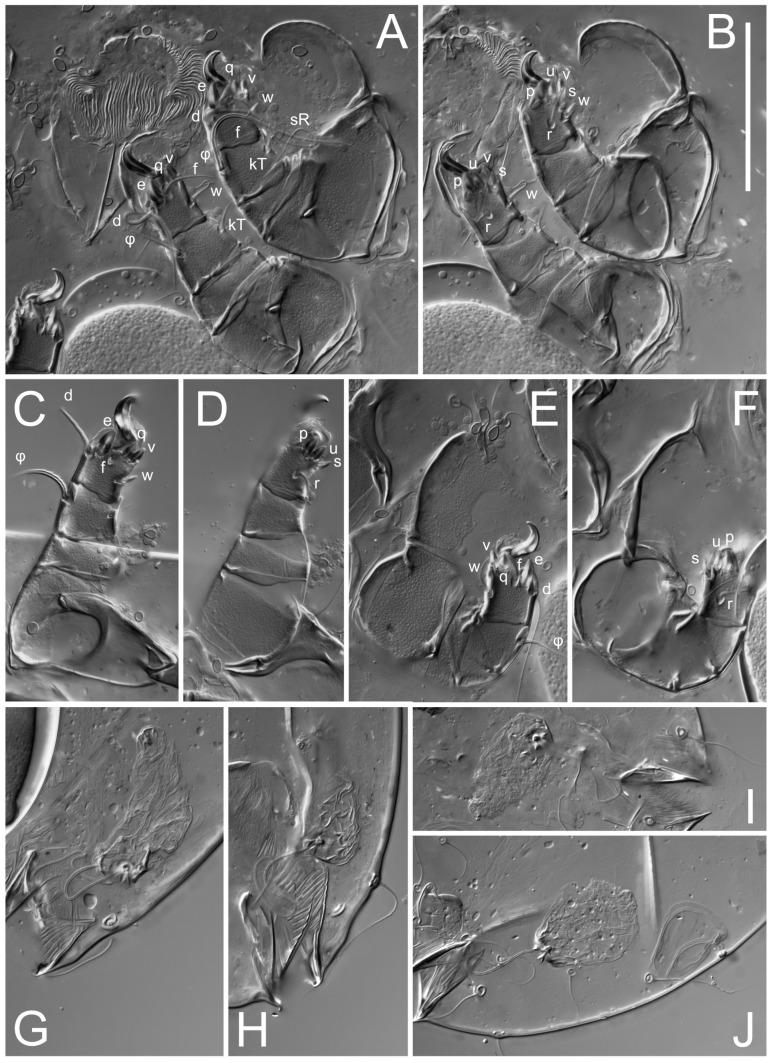
*Thyreophagus ais* sp. n., female (holotype (**A**–**G**) and paratypes (**H**–**J**)), DIC images: (**A**)—legs III–IV, dorsolateral view; (**B**)—legs III–IV, ventrolateral view; (**C**)—leg III, dorsolateral view; (**D**)—leg III, ventrolateral view; (**E**)—leg IV, dorsolateral view; (**F**)—leg IV, ventrolateral view; (**G**–**J**)—spermatheca. Scale bar: 50 μm.

**Female** (Figure 1, Figure 2, Figure 3, Figure 4 and Figure 5). Idiosoma elongate, 600 × 250 (holotype), 400–620 × 120–230 (paratype, *n* = 4), 2.4 (2.6–3.3, *n* = 4)-times longer than wide. Idiosomal cuticle smooth. Subcapitular setae (*h*) long, widened basally; palp tibial setae (*a*), lateral dorsal palp tibial setae (*sup*), dorsal palp tarsal seta (*cm*) filiform; supracoxal seta *elcp* absent; terminal palp tarsal solenidion ω short; external part of eupathidium *ul*’’ dome-shaped; terminal eupathidium *ul*’ not observed. Prodorsal sclerite 105 (70–94, *n* = 4) long, 96 (57–82, *n* = 4) wide, 1.1 (1.1–1.2, *n* = 4)-times longer than wide, both smoothly punctated and very finely longitudinally striated, except in anterior 1/5 (smoothly punctate) and posterior 1/4 (ornamented with distinct pattern of broken striae arranged in a triangle). Prodorsal sclerite with setae *vi* situated at anterior part of shield (bases touching but separated from each other), rounded anterolateral incisions, and elongate midlateral incisions (corresponding to insertion points of setae *ve*).

Grandjean’s organ (GO) expanded into 13 membranous finger-like extensions. Supracoxal seta (*scx*) smooth, sword-shaped, widened, and flattened, tapering at tip. Idiosomal setae (*vi*, *se*, *c*_p_, *d*_2_, *e*_2_, *h*_1_, *h*_2_, *h*_3_, *ps*_3_) smooth, filiform, and short; opisthosomal gland openings slightly anteriad of setal bases *e*_2_. One pair of fundamental cupules (*ia*) present (other cupules not observed).

Ventral idiosoma with four pairs of coxal setae (*1a*, *3a*, *4a*, and *4b*) and one pair of genital setae (*g*). Shape of coxal sclerites as shown in Figure 1B, Figure 3C,D, and Figure 5E,F. Ovipore situated between coxal fields III and IV; genital valves of ovipore shaped as an inverted Y; epigynal and medial apodemes well-developed. Genital papillae medium-sized, their diameters approximately 5–6-times shorter than the length of genital setae. Anal opening on posterior margin of idiosoma, mostly ventral, with substantial portion situated terminally and dorsally. Copulatory tube weakly developed. Canal of spermatheca long, slender, and uniform in diameter (not widened at entrance to spermatheca), forming a small, well-sclerotized, cup-shaped atrium. Spermatheca simple, with small sclerotized base and small, paired, Y-shaped sclerites of oviducts.

Legs short. Trochanters I–III each with long, filiform seta, *pR* I–II, *sR* III; trochanter IV without setae. Femoral setation 1-1-0-1; setae *vF* I–II and *wF* IV long, filiform. Genual setation 2-2-0-0; setae *mG* and *cG* I–II long, filiform; seta *nG* III absent. Tibial setation 2-2-1-1; setae *hT* I-II filiform; setae *gT* I–II and *kT* III–IV elongated, somewhat spiniform. Tarsal setation 10-10-10-10; all pretarsi with hooked empodial claws arising from tarsal apices and short paired condylophores. Tarsus I with 10 setae; *ra*, *la*, *f*, and *d* filiform; *e*, *u*, *v*, *p*, *q* spiniform (*p* and *q* distinctly shorter than *u* and *v*); *s* flattened, button-shaped; setae *wa* absent. Tarsal II setation similar to that of tarsus I, except proral setae (*p*, *q*) represented by small triangular rudiments (Figure 4J). Tarsus III with 10 setae; *f*, *d*, *r* filiform; *e*, *w*, *s*, *u*, *v*, *p*, *q* spiniform. Tarsus IV similar to tarsus III, except *w* filiform. Solenidion ω_1_ on tarsus I cylindrical, with clavate apex, slightly curved; solenidion ω_1_ on tarsus II simple, cylindrical, with clavate apex, not bent, shorter than ω_1_ on tarsus I. Solenidion ω_2_ on tarsus I shorter than ω_1_, cylindrical, with rounded apex, slightly widened at tip, situated slightly anterior and external to ω_1_. Solenidion ω_3_ on tarsus I cylindrical, with rounded apex, subequal to ω_1_, longer than ω_2_. Famulus (ε) of tarsus I wide, spiniform, with broadly rounded apex, widest at middle. Solenidia φ of tibiae I–III elongate, tapering, well-extended beyond apices of respective tarsi with ambulacra; solenidion φ IV shorter, shorter than tarsus IV (with ambulacra). On genu I, solenidion σ’ elongate, tapering, slightly not reaching bases of φ I, about 1.8-times longer than σ’’; σ’’ I slightly wider than σ’, about half the length of tibia I. On genu II, solenidion σ very short and somewhat conical. Solenidion σ of genu III absent.

**Male.** Absent.

**Figure 6:**
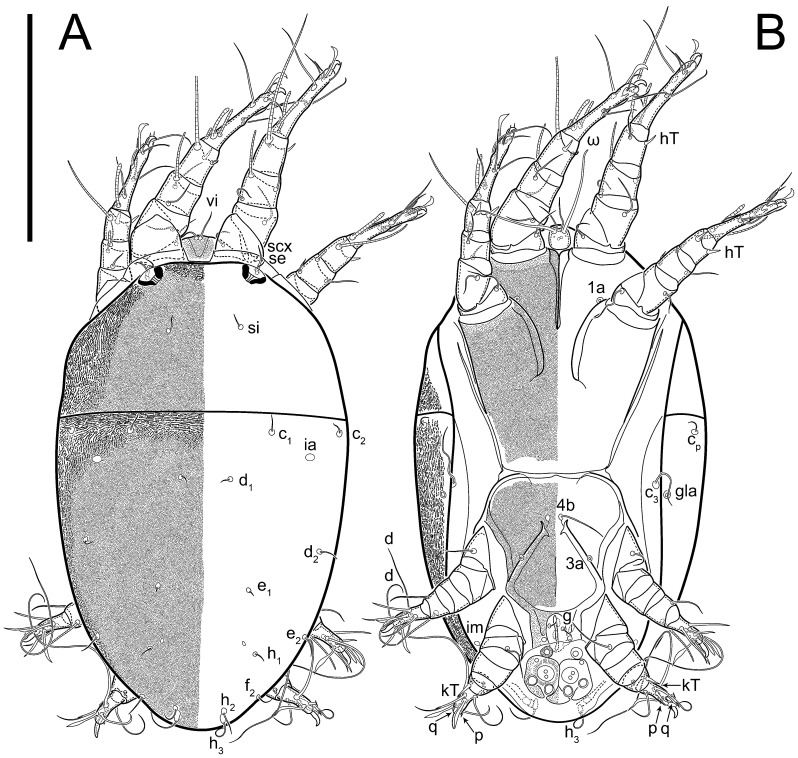
*Thyreophagus ais* sp. n., heteromorphic deutonymph: (**A**)—dorsal view; (**B**)—ventral view. Scale bar: 100 μm.

**Figure 7:**
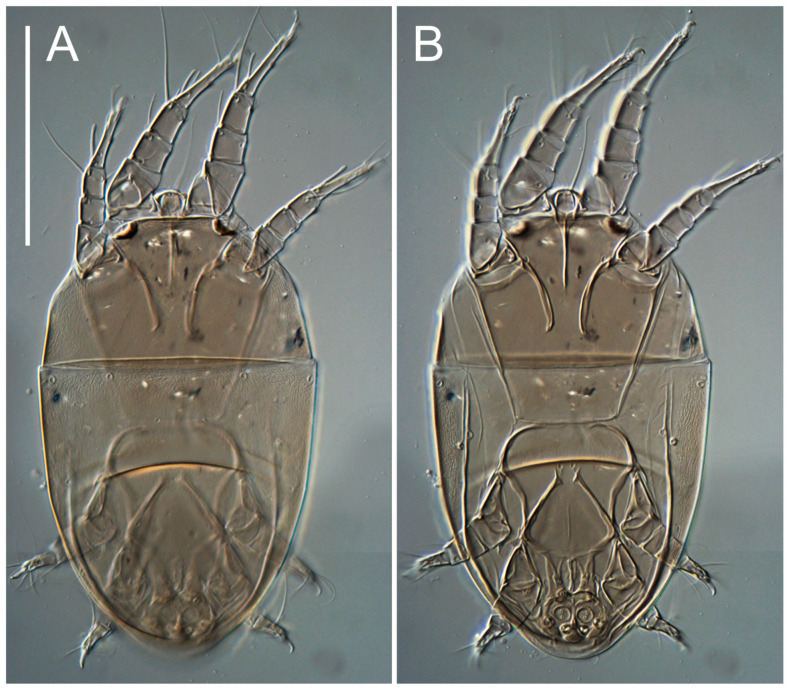
*Thyreophagus ais* sp. n., heteromorphic deutonymph, DIC images: (**A**)—dorsal view; (**B**)—ventral view. Scale bar: 100 μm.

**Figure 8:**
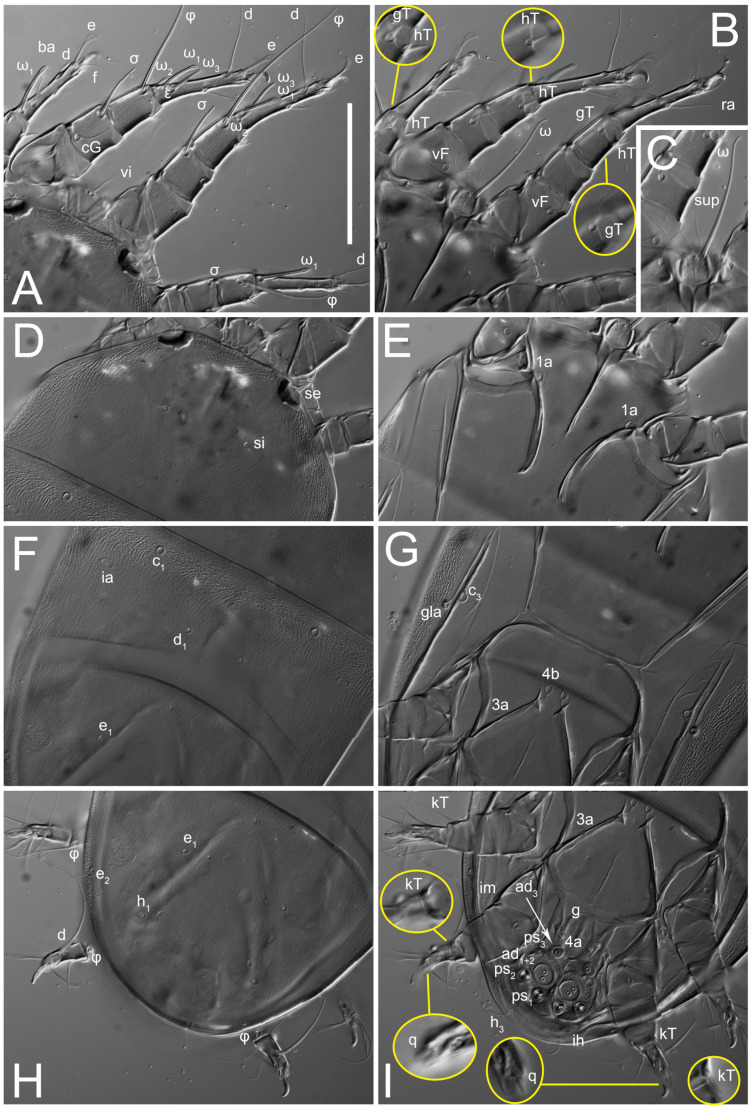
*Thyreophagus ais* sp. n., heteromorphic deutonymph, DIC images: (**A**,**D**,**F**,**H**)—dorsal views; (**B**,**E**,**G**,**I**)—ventral views; (**C**)—gnathosoma, ventral view. Scale bar: 50 μm.

**Heteromorphic deutonymph** (Figure 6, Figure 7 and Figure 8). Body elongate, 1.53–1.84-times longer than wide (*n* = 4), widest in sejugal region; idiosomal length 210–240, *n* = 4 width 130–150, *n* = 4. Gnathosoma short, subcapitulum and palp fused, bearing apical palpal solenidia (ω) and filiform apicodorsal setae (*sup*); setae *h* absent from subcapitular remnant, their positions marked by refractile spots.

Dorsum. Propodosoma and hysterosoma each covered by smoothly punctate shields; distinct linear pattern present on anterior and lateral sides of propodosomal sclerite and hysterosomal shield. Anterior propodosoma with internal vertical setae (*vi*) (bases separated) and a single sclerite. A pair of lateral ocelli present on propodosoma, widely separated from each other (distance 35–42, *n* = 4); lenses and pigmented spots present; maximal diameter of lenses 12–14, *n* = 4. External vertical setae (*ve*) absent; external scapular setae *se* situated just below eye lenses; internal scapular setae (*si*) distinctly posterior and medial to external scapulars (*se*). Supracoxal setae of legs I (*scx*) filiform, with extended base, satiated below *se*. Sejugal furrow well-developed. Propodosomal sclerite 65–80, *n* = 4, hysterosomal shield 139–160, *n* = 4, ratio hysterosomal shield/propodosomal sclerite = 2.0–2.13. Hysterosomal shield with 11 pairs of simple, filiform setae (*c*_1_, *c*_2_, *c*_p_, *d*_1_, *d*_2_, *e*_1_, *e*_2_, *f*_2_, *h*_1_, *h*_2_, *h*_3_), setae *h*_3_ distinctly longer than others. Opisthonotal gland openings (*gla*) ventral, situated on hysterosomal shield, slightly posterior to setae *c*_3_. Of four fundamental pairs of cupules, three pairs observed: *ia* posteriomediad of setae *c*_2_, *im* ventral, lateral to trochanter IV, *ih* ventral, lateral to posterior portion of attachment organ.

Venter. Coxal fields sclerotized, smoothly punctate. Anterior apodemes of coxal fields I fused forming sternum; sternum not reaching posterior border of sternal shield by distance exceeding its length. Posterior border of sternal shield not sclerotized. Anterior apodemes of coxal fields II curved medially. Posterior apodemes of coxal fields II weakly developed, thin. Sternal and ventral shield adjacent. Anterior apodemes of coxal fields III free, connected by thin transverse sclerotization. Posterior medial apodeme present in area of coxal fields IV, well-separated from anterior apodemes IV and genital opening. Posterior apodemes IV absent. Subhumeral setae (*c*_3_) filiform, situated on ventral surface between legs II–III, adjacent to region separating sternal and ventral shields. Coxal setae *1a*, *3a* reduced, represented by very small structures each within an alveolus. Coxal setae (*4b*) filiform, situated at tips anterior to coxal apodemes IV; *4a* in form of small, rounded conoids. Progenital region in posterior portion of coxal fields IV; genital opening elongate; two pairs of genital papillae within genital atrium; genital papillae two-segmented, with rounded apices; genital setae (*g*) filiform, situated laterad of genital opening. Attachment organ posterior to coxal fields IV. Anterior suckers (*ad*_3_) round, median suckers (*ad*_1+2_) distinctly larger, with paired vestigial alveoli; pair of small refractile spots anterolateral to median suckers (*ps*_3_); lateral conoidal setae of attachment organ (*ps*_2_) situated distinctly posterior to line joining centers of median suckers, slightly anterior conoids (*ps*_1_) and slightly posterior to median suckers (*ad*_1+2_); anterior and posterior lateral and posterior median cuticular conoids well-developed; anus situated between anterior suckers (*ad*_3_).

Legs. Legs elongate, all segments free. Trochanters I–III each with long, filiform seta, *pR* I–II, *sR* III. Femoral setation 1-1-0-1; setae *vF* I–II and *wF* IV long, filiform. Genual setation 2-2-0-0; setae *mG* and *cG* I–II filiform, seta *nG* III absent. Tibial setation 2-2-1-1; setae *hT* I somewhat spiniform; setae *gT* I filiform (Figure 8B); setae *gT* and *hT* II spiniform; setae *kT* III filiform; setae *kT* IV spiniform. Tarsal setation 7-8-8-8. All pretarsi consisting of hooked empodial claws arising from tarsal apices attached to short, paired condylophores within tarsal apices. Tarsus I with six filiform setae (*ra*, *la*, *p*, *q*, *d*, and *f*), setae *d* elongate, their bases situated at level anterior to bases of setae *ra* and *la*; one spoon-shaped seta *e*; setae *s* alveolar, setae *wa*, *aa* and *ba* I absent; tarsus II similar to tarsus I except seta *ba* present and filiform, close to ω_1_. Tarsus III with eight setae (*w*, *r*, *s*, *p*, *q*, *e*, *f*, *d*) smooth; all setae, except *d* III more or less foliate; seta *d* equal to or longer than leg III. Tarsus IV similar to tarsus III, except setae *q* and *p* short, spiniform, seta *r* longer, filiform, seta *w* filiform and with distinct prong, seta *d* distinctly longer than legs IV. Solenidia ω_1_ on tarsi I–II cylindrical, with slightly clavate apices; ω_3_ on tarsus I slightly shorter than ω1, with rounded apex, positioned slightly anterior to ω_1_; ω_1_ and ω_3_ separated by bulbous famulus (ε); solenidion ω_2_ of tarsus I slightly widened apically, situated somewhat more basal and posterior to ω_1_ + ε+ω_3_ group; solenidia φ of tibiae I–III elongate, tapering; φ I longer than tarsus I; φ II shorter than tarsus II; φ III reaching tip of tarsus III without ambulacrum; φ IV short; σ of genu I elongate, tapering slightly, nearly reaching tip of tibia I; σ of genu II shorter, cylindrical, not reaching midlength of tibia II; σ of genu III absent.

**Diagnosis**

**Figure 9:**
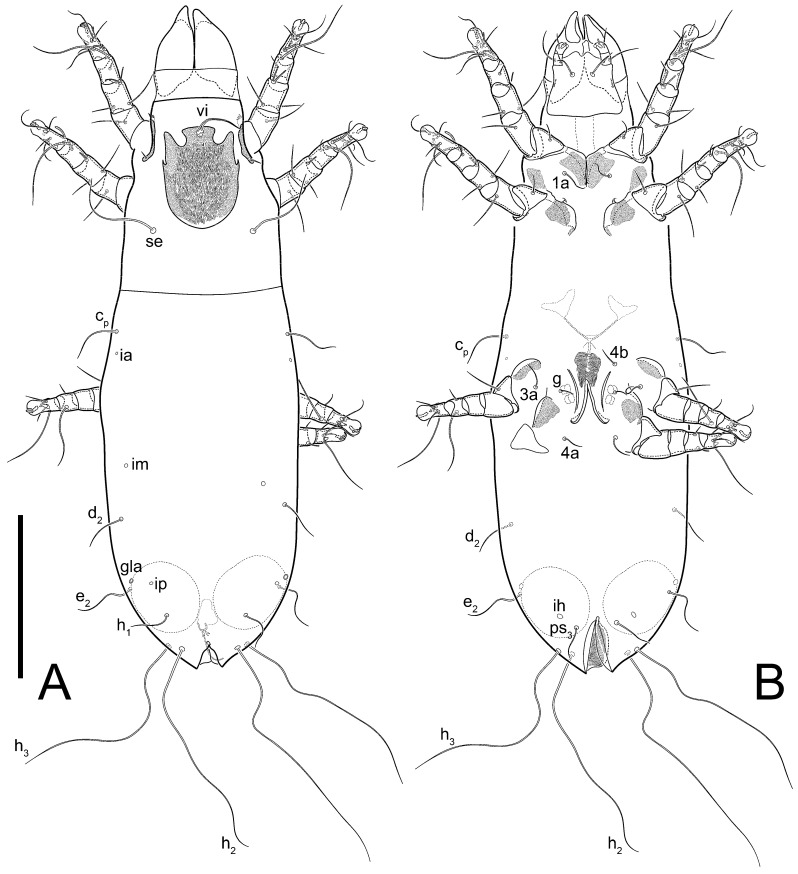
*Thyreophagus hobe* sp. n., female (holotype): (**A**)—dorsal view; (**B**)—ventral view. Scale bar: 100 μm.

Female. *Thyreophagus ais* is close to *Th. calusorum*, *Th. mauritianus* (Fain, 1982), *Th. gallegoi* Portus and Gomez, 1979 and *Th. vermicularis* Fain and Lukoschus, 1982 in having a very short solenidion σ II. *Th. ais* is similar to *Th. calusorum* and *Th. mauritianus* by the shape of solenidion σ II, which has convex sides (sides straight and not convex in *Th. gallegoi* and *Th. vermicularis*), but it differs from *Th. calusorum* by the following: sclerites of oviducts are Y-shaped (V-shaped in *Th. calusorum*); the canal of spermatheca at the entrance to spermatheca is not widened (Figure 2I) vs. widened in *Th. calusorum* [8] (Figure 9); the canal of spermatheca forms a small, well-sclerotized, cup-shaped atrium (Figure 2I and Figure 5G–J) vs. canal uniform in appearance, atrium absent [8] (Figure 9); bases of setae *vi* are touching but separated (in common area in *Th. calusorum*). *Th. ais* differs from *Th. mauritianus* as follows: the cuplike portions of the sclerites of oviducts are subequal to their stems (Figure 5G–J vs. shorter than stems in *Th. mauritianus* [8] (Figure 31B–D); solenidion σ’ is distinctly longer than σ’’ (subequal in *Th. mauritianus*); solenidion ω_1_ II is three-times longer than its width (five-times longer in *Th. mauritianus*); and solenidion φ IV is nearly reaching the bases of setae *d* IV (reaching the middle of tarsus IV in *Th. mauritianus*). *Th. ais* differs from *Th. gallegoi* by the absence of linear sclerites near the typical sclerites of oviducts (present in *Th. gallegoi*) and from *Th. vermicularis* by the Y-shaped paired sclerites of oviducts, which are at least three-times longer than their width (V-shaped, more than five-times longer than their width in *Th. vermicularis*).

Heteromorphic deutonymph. *Th. ais* is very similar to *Th. calusorum*, but differs by short, spiniform setae *q* and *p* IV (foliate in *Th. calusorum*), setae *d* III are longer than leg III (shorter in *Th. calusorum*), *d* IV distinctly longer than leg IV (subequal to leg IV in *Th. calusorum*). Setae *hT* I and *kT* IV are always spiniform in *Th. ais*, but it can be variable, either spiniform or filiform in *Th. calusorum* (Klimov et al., 2022).

**Figure 10:**
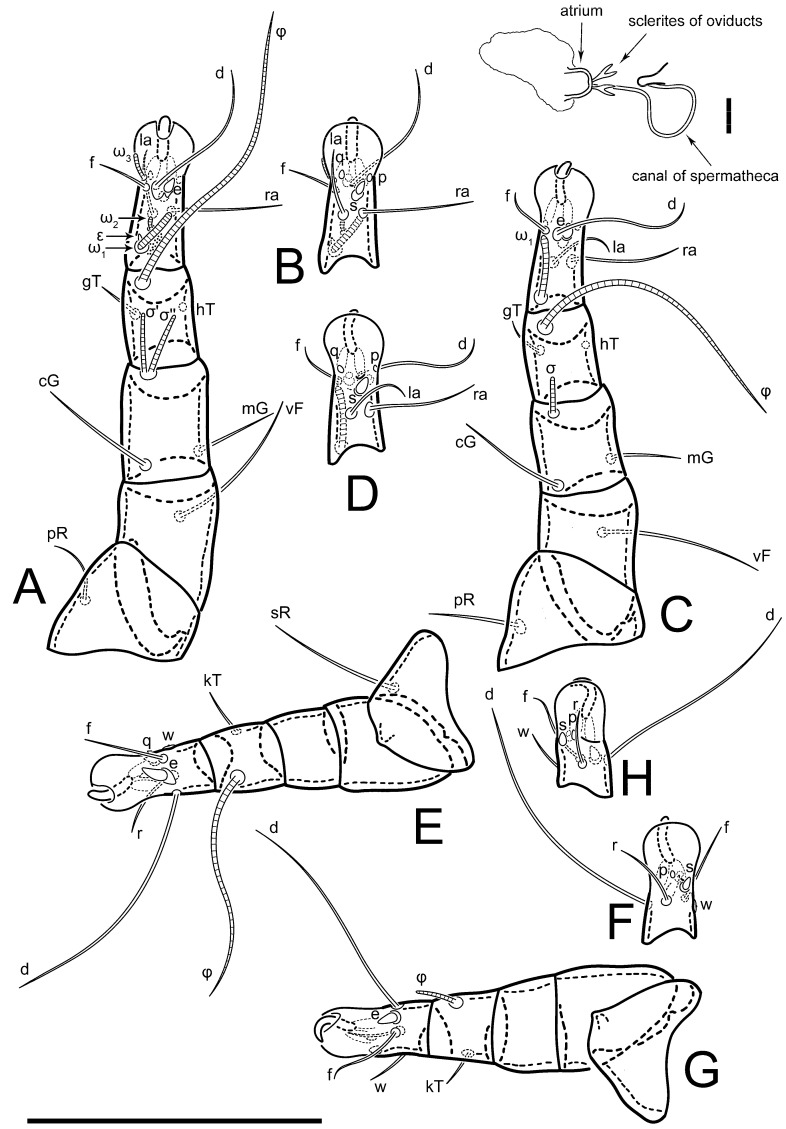
*Thyreophagus hobe* sp. n., female (holotype): A—leg I, dorsal view; B—tarsus I, ventral view; C—leg II, dorsal view; D—tarsus II, ventral view; E—leg III, dorsolateral view; F—tarsus III, ventrolateral view; G—leg IV, dorsolateral view; H—tarsus IV, ventrolateral view; I—spermatheca. Scale bar: 50 μm.

**Figure 11:**
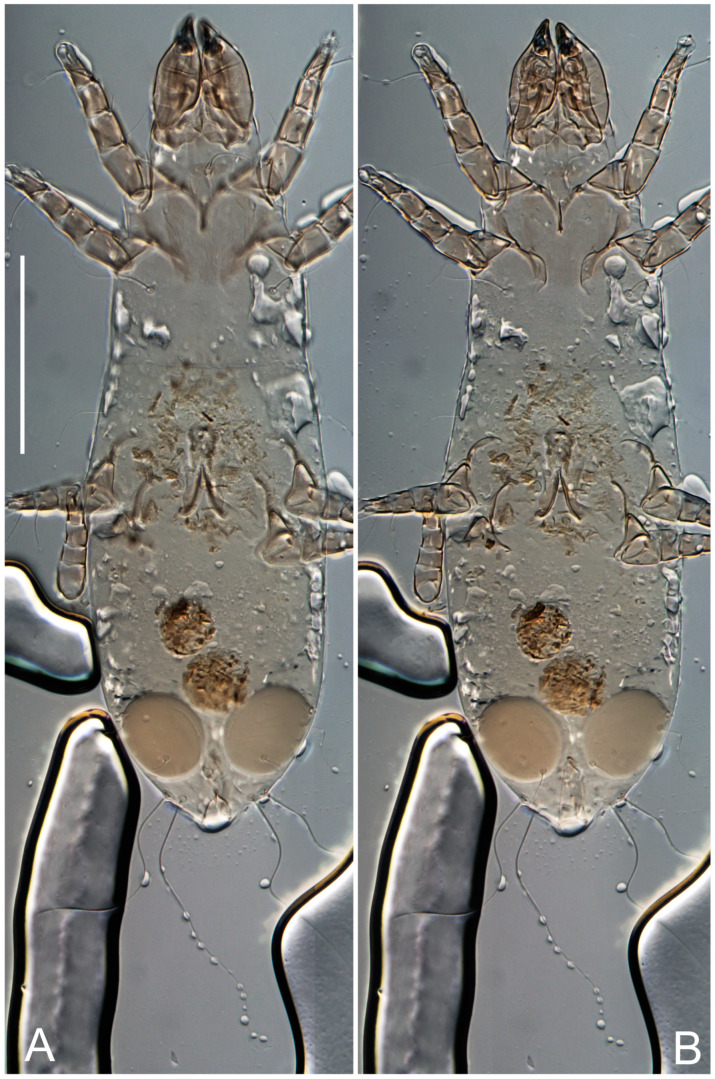
*Thyreophagus hobe* sp. n., female (holotype), DIC images: (**A**)—dorsal view; (**B**)—ventral view. Scale bar: 100 μm.

**Figure 12:**
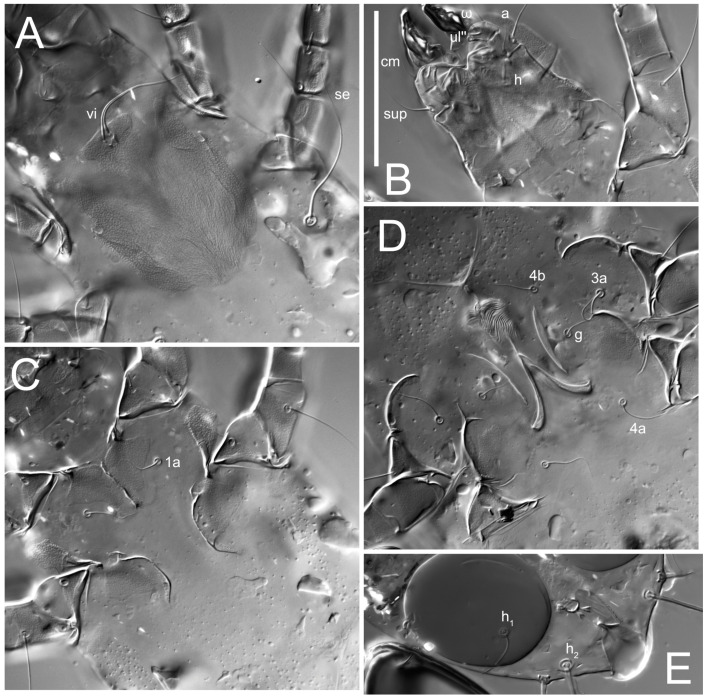
*Thyreophagus hobe* sp. n., female (holotype), DIC images: (**A**)—prodorsal shield; (**B**)—gnathosoma, ventral view; (**C**)—coxal fields I–II; (**D**)—ovipore and coxal fields III–IV; (**E**)—spermatheca. Scale bar: 50 μm.

**Figure 13:**
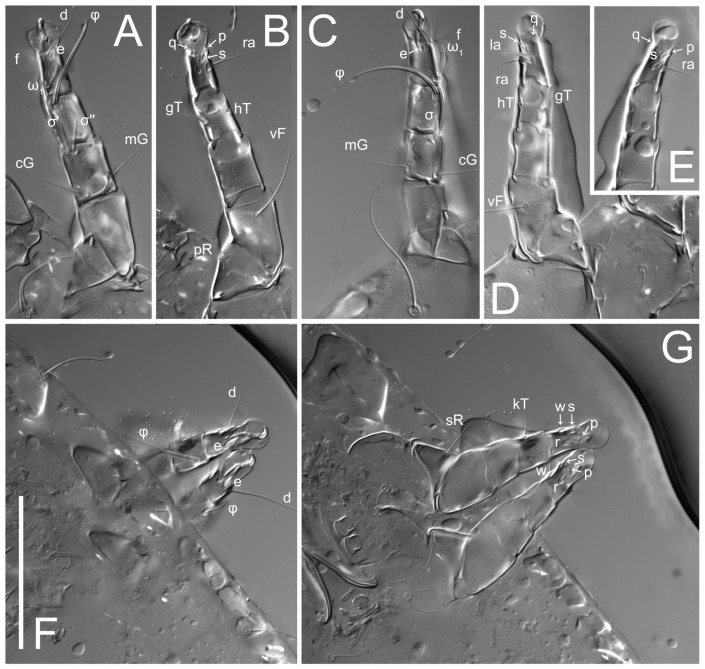
*Thyreophagus hobe* sp. n., female (holotype), DIC images: (**A**)—leg I, dorsal view; (**B**)—leg I, ventral view; (**C**)—leg II, dorsal view; (**D**,**E**)—leg II, ventral view; (**F**)—legs III–IV, dorsal view; (**G**)—legs III–IV, ventral view. Scale bar: 50 μm.

*Thyreophagus hobe* Klimov, Kolesnikov, Demard, Vangansbeke, sp. n.

(Figure 9, Figure 10, Figure 11, Figure 12 and Figure 13).

urn:lsid:zoobank.org:pub:7C7FEAA7-EB57-4A12-A489-196D2BEEA5D9.

**Type material**. Holotype: female—USA: Florida, Fort Pierce, branches on ground in a small, wooded area, subcortical, stick3, 27°25′34.5″ N 80°24′22.7″ W, 12 October 2020, Emilie Demard, BMOC 20-0101-008. Culture maintained until 26 February 2021 but then was accidentally lost (no specimens from these cultures were preserved).

**Depository**. Holotype—University of Michigan, Museum of Zoology, Ann Arbor, Michigan, USA.

**Etymology**. The new species is named after the Hobe Indians who lived in the eastern coastal area of central Florida (between St. Lucie and Jupiter inlets), but went extinct after the arrival of European colonizers [27]. The men of the Hobe Indians had long hair ^6^, which is reminiscent of the long dorsal setae of *Thyreophagus hobe*.

**Habitat**. *Thyreophagus hobe* lives under the bark of small fallen branches found in wooded areas. These branches are typically in the initial stages of decomposition, with approximately 80% of their natural sapwood retaining a white color, and 20% displaying brown discoloration, indicating the ongoing decomposition process.

**Description**

**Female** (Figure 9, Figure 10, Figure 11, Figure 12 and Figure 13). Idiosoma elongate, 350 × 120 (holotype), 2.9-times longer than wide. Idiosomal cuticle smooth. Subcapitular setae (*h*) long, widened basally; palp tibial setae (*a*), lateral dorsal palp tibial setae (*sup*), dorsal palp tarsal seta (*cm*) filiform; supracoxal seta *elcp* present, slightly widened basally; terminal palp tarsal solenidion ω short; external part of terminal eupathidium *ul*’’ dome-shaped; terminal eupathidium *ul*’ not observed. Prodorsal sclerite 63 long, 46 wide, 1.4-times longer than wide, with setae *vi* (situated at anterior part of shield, bases in common area of unsclerotized cuticle), rounded anterolateral incisions, and elongate midlateral incisions (corresponding to insertion points of setae *ve*); shield punctate, 3/4 of posterior central region with linear pattern. Grandjean’s organ (GO) expanded anteriorly into membranous finger-shaped extensions (exact number cannot be observed on the single specimen). Supracoxal seta (*scx*) smooth, sword-shaped, widened and flattened, tapering at tip. Idiosomal setae (*vi*, *se*, *c*_p_, *d*_2_, *e*_2_, *h*_1_, *h*_2_, *h*_3_, *ps*_3_) smooth, filiform, setae *h*_2_ and *h*_3_ very long (2.3-times longer than length of prodorsal shield); opisthosomal gland openings slightly anteriad of setal bases *e*_2_. Four pairs of fundamental cupules (*ia*, *im*, *ip*, *ih*) present.

Ventral surface of idiosoma with four pairs of coxal setae (*1a*, *3a*, *4a*, and *4b*) and one pair of genital setae (*g*), seta *4b* unpaired in holotype (abnormality). Shape of coxal sclerites as shown in Figure 1B and Figure 4C,D. Genital region between coxal fields III and IV; genital valves shaped in an inverted Y; epigynal and medial apodemes well-developed. Genital papillae medium-sized, their diameter approximately 5–6-times shorter than length of genital setae. Anal opening situated on posterior margin of idiosoma, mostly ventral, with substantial portion positioned terminally and dorsally. Copulatory tube weakly developed. Canal of spermatheca medium-sized, slender, uniformly wide, not widening at entrance to spermatheca. Spermatheca simple, with sclerotized, dome-shaped atrium (length and width subequal), small, paired Y-shaped sclerites of oviducts, and large sperm storage sac.

Legs short, all segments free. Trochanters I–III each with long, filiform seta, *pR* I–II, *sR* III; trochanter IV without setae. Femoral setation 1-1-0-0; setae *vF* I–II long, filiform, *wF* IV absent. Genual setation 2-2-0-0; setae *mG* and *cG* I–II long, filiform; seta *nG* III absent. Tibial setation 2-2-1-1; setae *hT* I-II represented by alveoli; setae *gT* I–II and *kT* III–IV elongated, filiform. Tarsal setation 8-8-8-8; all pretarsi have hooked empodial claws arising from tarsal apices and attached to short paired condylophores. Tarsus I with eight setae; *ra*, *la*, *f*, and *d* filiform; *e*, *s*, *p*, and *q* spiniform, small; *u* and *v* absent (represented by weakly developed rounded structures), setae *wa* absent. Tarsus II setation similar to that of tarsus I. Tarsus III with eight setae; *f*, *d*, *r* filiform; *e* and *s* spiniform; *p*, *q*, *u*, and *v* similar to these of tarsus I–II; seta *w* flattened, button-shaped. Tarsus IV similar to tarsus III, except *w* filiform. Solenidion ω_1_ on tarsus I cylindrical, with clavate apex, slightly curved; solenidion ω_1_ on tarsus II simple, cylindrical, with clavate apex, not bent, longer than ω_1_ on tarsus I. Solenidion ω_2_ on tarsus I shorter than ω_1_, cylindrical, with rounded apex, slightly expanded at tip, situated slightly anterior and external to ω_1_. Solenidion ω_3_ on tarsus I cylindrical, with rounded apex, shorter than ω_1_ I. Famulus (ε) of tarsus I spiniform, with pointed apex. Solenidia φ of tibiae I–III elongate, tapering, well extending beyond apices of respective tarsi with ambulacra; solenidion φ IV shorter than tarsus IV (with ambulacra). Genual solenidia σ’ and σ’’ I elongate, tapering, subequal, slightly not reaching bases of φ I. Genual solenidion σ II 7–9-times longer than its width, with rounded tip. Genual solenidion σ III absent.

**Male.** Absent.

**Heteromorphic deutonymph**. Unknown.

**Diagnosis**

Female. *Thyreophagus hobe* differs from all known species of *Thyreophagus* by the absence of femoral setae *wF* IV and unguinal setae *u*, *v* I–IV. Like *Th. australis* Clark, 2009, *Th. hobe* has very long setae *h*_2_ and *h*_3_ (2.3-times longer than the length of the prodorsal shield in *Th. hobe* (2.5–2.8-times longer than the length of the prodorsal sclerite in *Th. australis*), but in *Th. hobe*, setae *h*_1_ five-times shorter than *h*_2_ and *h*_3_ (vs. *h*_1_, *h*_2_, and *h*_3_ are subequal in *Th. australis*).

**Figure 14:**
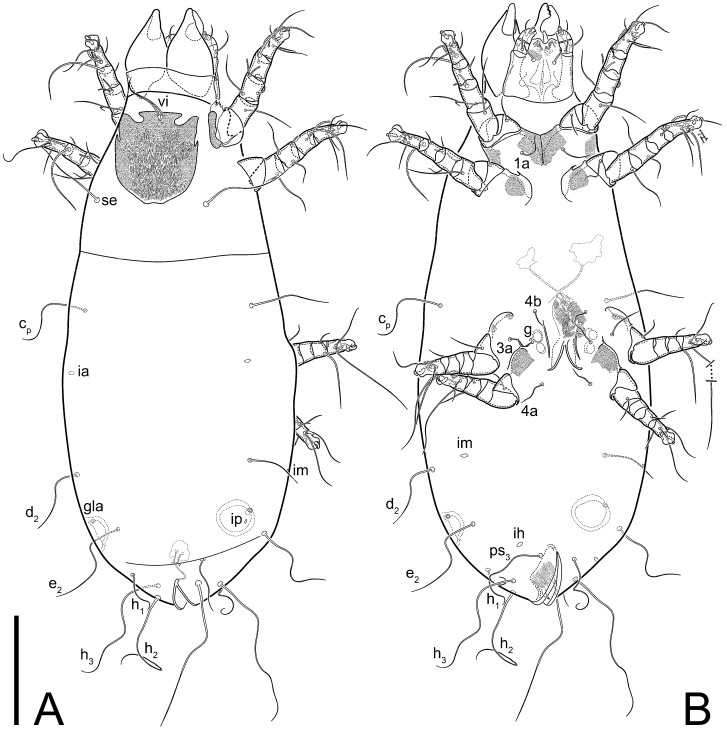
*Thyreophagus ojibwe* sp. n., female (holotype): (**A**)—dorsal view; (**B**)—ventral view. Scale bar: 100 μm.

**Figure 15:**
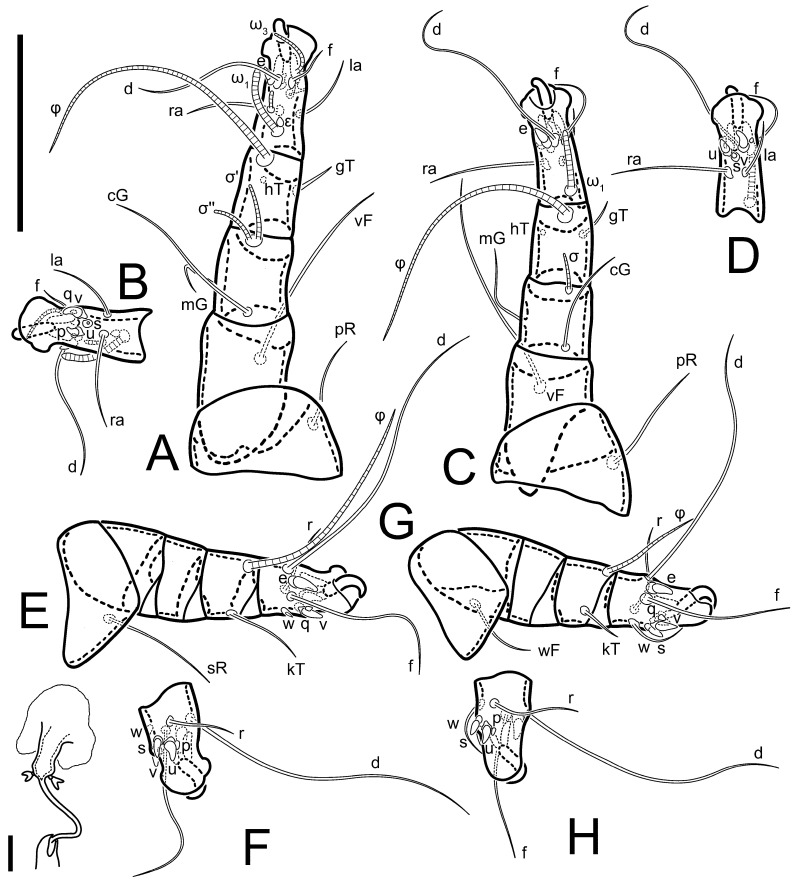
*Thyreophagus ojibwe* sp. n., female (holotype): A—leg I, dorsal view; B—tarsus I, ventral view; C—leg II, dorsal view; D—tarsus II, ventral view; E—leg III, dorsolateral view; F—tarsus III, ventrolateral view; G—leg IV, dorsolateral view; H—tarsus IV, ventrolateral view; I—spermatheca. Scale bar: 50 μm.

**Figure 16:**
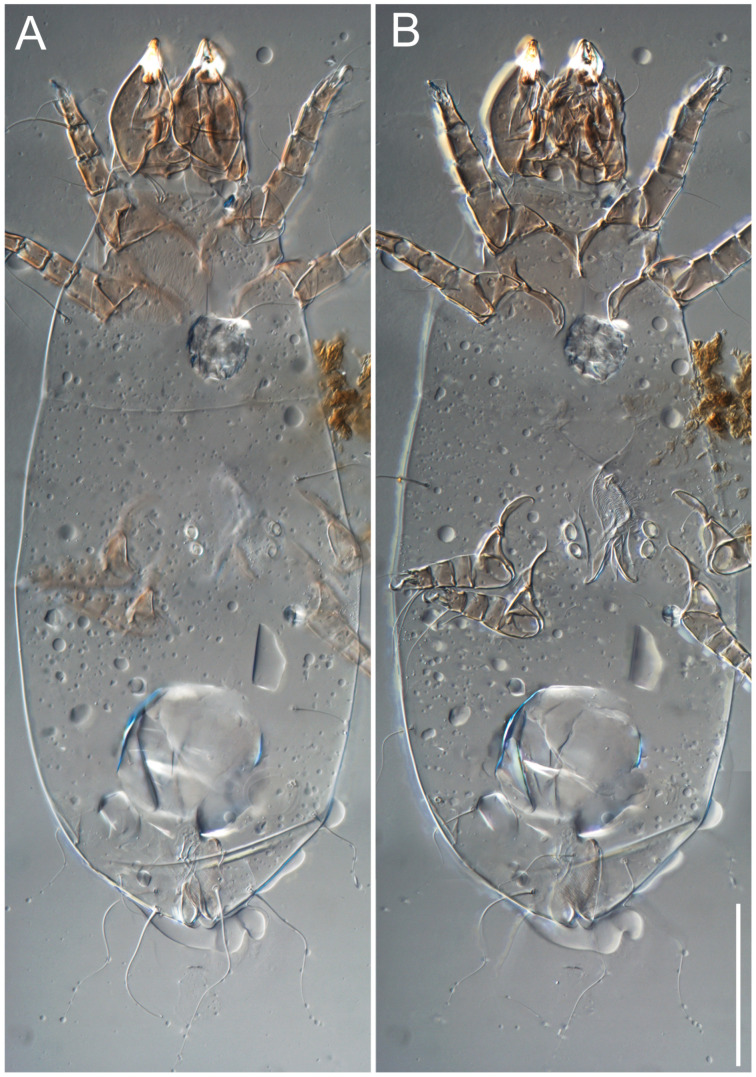
*Thyreophagus ojibwe* sp. n., female (holotype), DIC images: (**A**)—dorsal view; (**B**)—ventral view. Scale bar: 100 μm.

**Figure 17:**
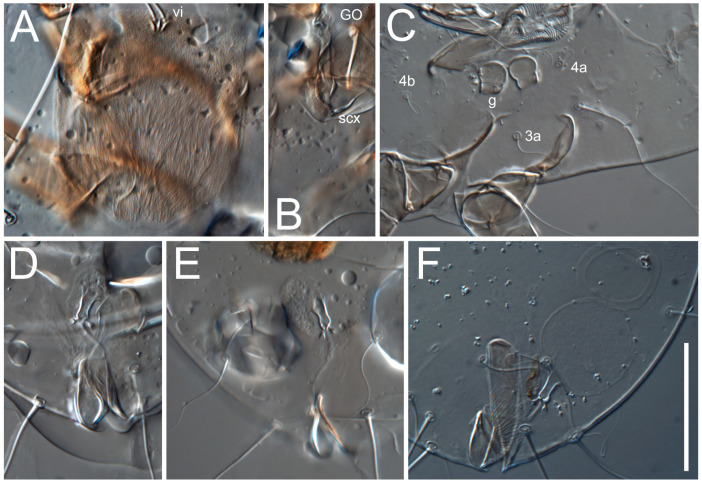
*Thyreophagus ojibwe* sp. n., female (holotype (**D**) and paratype (**E**,**F**)), DIC images: (**A**)—prodorsal shield; (**B**)—supracoxal sclerite and Grandjean’s organ; (**C**)—ovipore and coxal fields III–IV; (**D**–**F**)—spermatheca. Scale bar: 50 μm.

**Figure 18:**
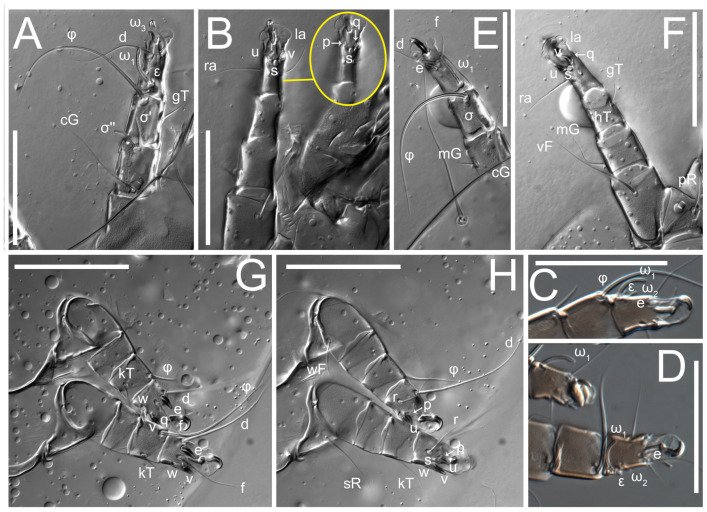
*Thyreophagus ojibwe* sp. n., female (holotype (**D**) and paratype (**E**,**F**)), DIC images: (**A**)—leg I, dorsal view; (**B**)—leg I, ventral view; (**C**)—leg I, lateral view; (**D**)—legs I–II, lateral view; (**E**)—leg II, dorsal view; (**F**)—leg II, ventral view; (**G**)—legs III–IV, dorsal view; (**H**)—legs III–IV, ventral view. Scale bar: 50 μm.

**Figure 19:**
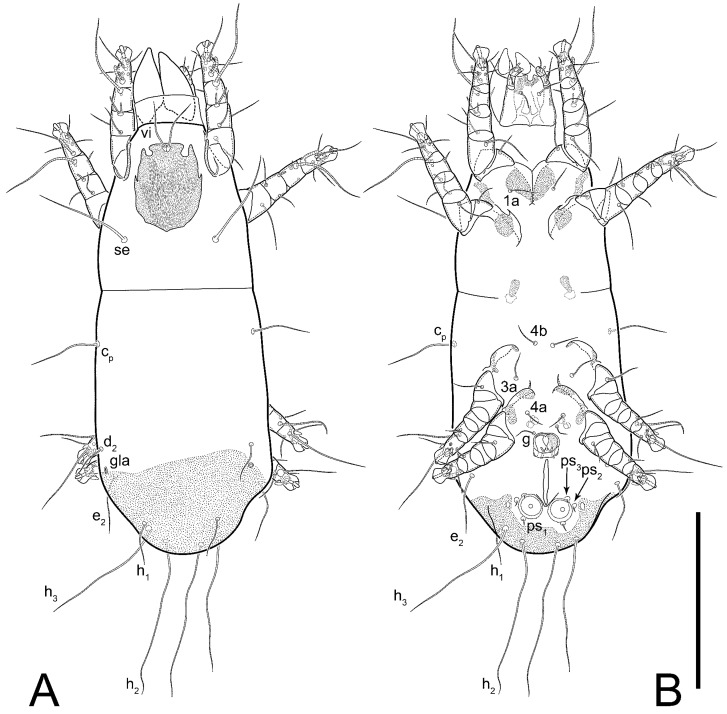
*Thyreophagus ojibwe* sp. n., male (paratype): (**A**)—dorsal view; (**B**)—ventral view. Scale bar: 100 μm.

**Figure 20:**
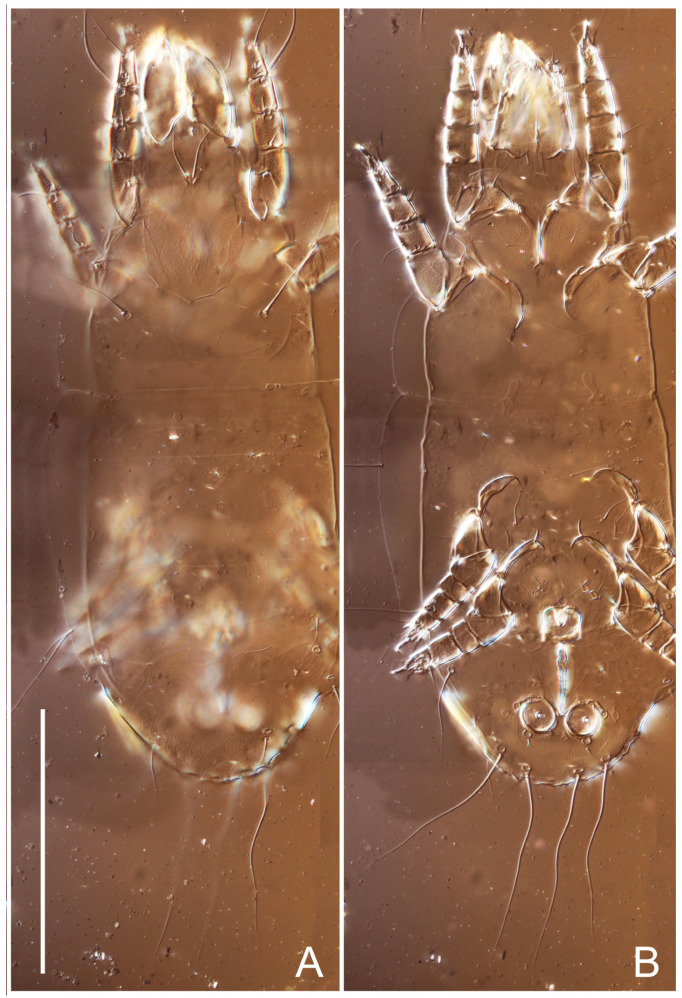
*Thyreophagus ojibwe* sp. n., male (paratype), DIC images: (**A**)—dorsal view; (**B**)—ventral view. Scale bar: 100 μm.

*Thyreophagus ojibwe* Klimov, Kolesnikov, Vangansbeke, sp. n.

(Figure 14, Figure 15, Figure 16, Figure 17, Figure 18, Figure 19 and Figure 20).

urn:lsid:zoobank.org:pub:7C7FEAA7-EB57-4A12-A489-196D2BEEA5D9.

**Type material**. Holotype female (f1), paratype female (f2)—USA: Michigan, Michigan State University Chestnut Plantation, *Castanea dentata* (Fagales: Fagaceae), branch on ground (tree1.stick1), 42°09′06.6″ N 84°25′33.2″ W, 23 November 2020, P. Klimov, BMOC 20-0101-010#slide1; 1 paratype female—same data, slide 2; 1 paratype female—same data, *C. dentata* blight tissues, BMOC 20-0101-012#slide3.

**Non-type material**. One male, two females—CANADA: Ontario C.E.F., Ottawa, under bark of dead willow twigs, *Salix* sp. (Malpighiales: Salicaceae), O. Peck, 1974 CNC788949 (Canadian National Collection) (studied as high-resolution images, provided by Fred Beaulieu, Canadian National Collection of Insects, Arachnids & Nematodes (CNC)).

**Depository**. Holotype, paratypes—University of Michigan, Museum of Zoology, Ann Arbor, Michigan, USA.

**Etymology**. The new species is named after the Ojibwe, North American indigenous people from what is currently southern Canada and Midwestern United States, including the state of Michigan.

**Habitat***. Thyreophagus ojibwe* lives under the bark of small fallen branches typically found around American chestnut trees. These branches are generally in the initial stages of decomposition. Additionally, mites were found on the blight-infected tissues, suggesting that these mites can likely feed on this harmful fungus and potentially transmit hypoviruses.

**Description**

**Female** (Figure 14, Figure 15, Figure 16, Figure 17 and Figure 18). Idiosoma elongate, 460 × 200 (holotype), 420-450 × 180-200 (paratype, *n* = 3), 2.3 (2.3, *n* = 3)-times longer than wide. Idiosomal cuticle smooth. Subcapitular setae (*h*) long, widened basally; palp tibial setae (*a*), lateral dorsal palp tibial setae (*sup*), dorsal palp tarsal seta (*cm*) filiform; supracoxal seta *elcp* present, slightly widened basally; terminal palp tarsal solenidion ω short; external part of terminal eupathidium *ul*’’ dome-shaped; terminal eupathidium *ul*’ not observed. Prodorsal sclerite 85 (71–80, *n* = 3) long, 75 (60–68, *n* = 3) wide, 1.1 (1.1–1.2, *n* = 3)-times longer than wide, with setae *vi* (situated at anterior part of sclerite, bases touching but distinctly separated), rounded anterolateral incisions, and elongate midlateral incisions (corresponding to insertion points of setae *ve*); shield smoothly punctate, 3/4 of posterior central region with linear pattern. Grandjean’s organ (GO) with 8 membranous, finger-shaped extensions. Supracoxal seta (*scx*) smooth, sword-shaped, widened and flattened, tapering at tip. Idiosomal setae (*vi*, *se*, *c*_p_, *d*_2_, *e*_2_, *h*_1_, *h*_2_, *h*_3_, *ps*_3_) smooth, filiform; setae *h*_2_ and *h*_3_ long, 1.75-times longer than length of prodorsal sclerite; opisthosomal gland openings slightly anteriad of setal bases of *e*_2_. Four pairs of fundamental cupules (*ia*, *im*, *ip*, *ih*) present.

Ventral surface of idiosoma with four pairs of coxal setae (*1a*, *3a*, *4a*, and *4b*) and one pair of genital setae (*g*). Shape of coxal sclerites as shown in Figure 1B. Genital region between coxal fields III and IV; genital valves shaped as an inverted Y; epigynal and medial apodemes well-developed. Genital papillae large, their diameters approximately four-times shorter than length of genital setae. Anal opening on posterior margin of idiosoma, mostly ventral, with substantial portion positioned terminally and dorsally. Canal of spermatheca short, slender tube, uniformly wide, slightly expanded at entrance to spermatheca. Spermatheca with elongated, vase-shaped atrium (longer than width); paired Y-shaped sclerites of oviducts small (their stems short, shorter than atrium in its narrowest part); and large sperm storage sac.

Legs short, all segments free. Trochanters I–III each with long, filiform seta, *pR* I–II, *sR* III; trochanter IV without setae. Femoral setation 1-1-0-1; setae *vF* I–II and *wF* IV long, filiform. Genual setation 2-2-0-0; setae *mG* and *cG* I–II long, filiform; seta *nG* III absent. Tibial setation 2-2-1-1; setae *hT* I-II alveolar; setae *gT* I–II elongated, somewhat spiniform; setae *kT* III–IV filiform. Tarsal setation 10-10-10-10; all pretarsi with hooked empodial claws attached to short, paired condylophores. Tarsus I with 10 setae; *ra*, *la*, *f*, and *d* filiform; *e*, *u*, *v* spiniform; *p* and *q* represented by small triangular rudiments; *s* flattened, button-shaped; setae *wa* absent. Tarsus II setation similar to that of tarsus I, except seta *s* small, spiniform. Tarsus III with 10 setae, *f*, *d*, *r* filiform, *e*, *s*, *u*, *v* spiniform, *w* small spiniform, *p* and *q* represented by small triangular rudiments. Tarsus IV similar to tarsus III, except *w* filiform. Solenidia ω_1_ on tarsi I and II cylindrical, with clavate apices, slightly curved, almost reaching tip of tarsus (without ambulacra). Solenidion ω_2_ on tarsus I shorter than ω_1_, cylindrical, with rounded apex, slightly widened at tip, situated slightly anterior and external to ω_1_. Solenidion ω_3_ on tarsus I cylindrical, with rounded apex, shorter than ω_1_, longer than ω_2_. Famulus (ε) of tarsus I wide, spiniform, with broadly rounded apex, widest at middle. Solenidia φ of tibiae I–III elongate, tapering, well extending beyond apices of respective tarsi with ambulacra; solenidion φ IV shorter, almost reaching tip of tarsus IV (without ambulacra). Genual solenidia σ’ and σ’’ I elongate, tapering, subequal, slightly not reaching bases of φ I. Genual solenidion σ II 10-times longer than its width) and somewhat conical. Genual solenidion σ III absent.

**Male** (*n* = 1) (Figure 19 and Figure 20). Idiosoma elongate, 250 × 120, 2.1-times longer than wide. Idiosomal cuticle smooth. Gnathosoma as in female. Prodorsal sclerite 50 long, 38 wide, 1.3-times longer than wide, with setae *vi*, incisions and ornamented as in female.

Grandjean’s organ (GO) and supracoxal seta (*scx*) as in female. Idiosomal setae (*vi*, *se*, *c*_p_, *d*_2_, *e*_2_, *h*_1_, *h*_2_, *h*_3_) smooth, filiform, setae *h*_2_ and *h*_3_ long (1.8-times longer than length of prodorsal shield); opisthosomal gland openings slightly anteriad of setal bases *e*_2_. Four pairs of fundamental cupules (*ia*, *im*, *ip*, *ih*) present. Opisthonotal shield solid, whole, smoothly punctated; ventral part of shield extends to anal suckers.

Ventral surface of idiosoma with four pairs of coxal setae (*1a*, *3a*, *4a*, and *4b*) and one pair of genital setae (*g*). Shape of coxal sclerites shown in Figure 19. Genital region between coxal fields IV; arms of genital capsule rounded; aedeagus short, not protruding beyond anterior edge of supporting sclerite. Genital papillae medium-sized, their diameter approximately 4–5-times shorter than genital setae. Anal sucker rounded in outline. Setae *ps*_1-3_ very short.

Legs I–III as in female, except solenidion ω_3_ on tarsus I very short, truncated and solenidion σ’’ about two-times longer than σ’. Trochanter and genua IV without setae, femur IV with setae *wF* IV long, filiform, tibia IV with *kT* IV elongated, somewhat spiniform. Tarsus IV with 10 setae; *f*, *r*, *w* filiform; *d* and *e* represented by suckers; *u*, *v*, *p*, *q* spiniform; *s* flattened, button-shaped or minute, spiniform. Solenidion φ on tibia IV short and wider.

**Heteromorphic deutonymph**. Unknown.

**Diagnosis**

Female. *Th. ojibwe* is very similar to *Th. corticalis*, but differs as follows: setae *h*_2_ and *h*_3_ are 1.75-times longer than the length of the prodorsal sclerite (1.1–1.25 times in *Th. corticalis*); the diameter of the genital papillae is approximately four-times shorter than the length of genital setae (six-times shorter in *Th. corticalis*); the stem of the Y-shaped sclerites of oviducts is about 3–4-times shorter than the length of Y-shaped sclerites of oviducts (subequal in *Th. corticalis*); ventral apical spines *p* and *q* of tarsi III–IV are shorter than spines *u* and *v* III–IV (subequal in *Th. corticalis*).

Male. *Th. ojibwe* differs from *Th. corticalis* by the longer setae *h*_2_ and *h*_3_ (their length is 1.8-times longer than the length of prodorsal sclerite (1.1–1.25-times longer in *Th. corticalis*) and by spines *p* and *q* on tarsi III–IV, which are shorter than spines *u* and *v* (subequal in *Th. corticalis*).

*Thyreophagus potawatomorum* Klimov, Kolesnikov, Vangansbeke, sp. n.

(Figure 21, Figure 22, Figure 23, Figure 24, Figure 25, Figure 26, Figure 27, Figure 28, Figure 29, Figure 30, Figure 31, Figure 32, Figure 33 and Figure 34).

urn:lsid:zoobank.org:pub:7C7FEAA7-EB57-4A12-A489-196D2BEEA5D9.

**Type material**. Holotype female (f2) and one paratype female (f1)—USA: Michigan, Ann Arbor, Hansen Nature Area, stick 5, 42°16′03.8″ N 83°46′53.2″ W, 14 October 2020, P. Klimov, BMOC 20-0101-004#slide 1; four paratype females—same data (culture, harvested 26 February 2021), slide 2; six paratype females—same data, slide 3; two paratype HDNs—same data, slide 5; one paratype HDN—same data, slide 6; one paratype HDN—same data, slide 8.

**Non-type material**. Two DNs, one pharate DN—same data, slide 7.

**Depository**. Holotype, paratypes—University of Michigan, Museum of Zoology, Ann Arbor, Michigan, USA.

**Etymology**. This species is named after Potawatomi, native American people of the Great Plains, upper Mississippi River, and western Great Lakes region (including the state of Michigan).

**Habitat***. Thyreophagus potawatomorum* lives under the bark of small fallen branches of deciduous trees in wooded areas. These branches are typically in the initial stages of decomposition, with approximately 80% of their natural sapwood retaining a white color, and 20% displaying brown discoloration, indicating the ongoing decomposition process. Additionally, the bark usually exhibits boreholes from wood-boring beetles.

**Description**

**Figure 21:**
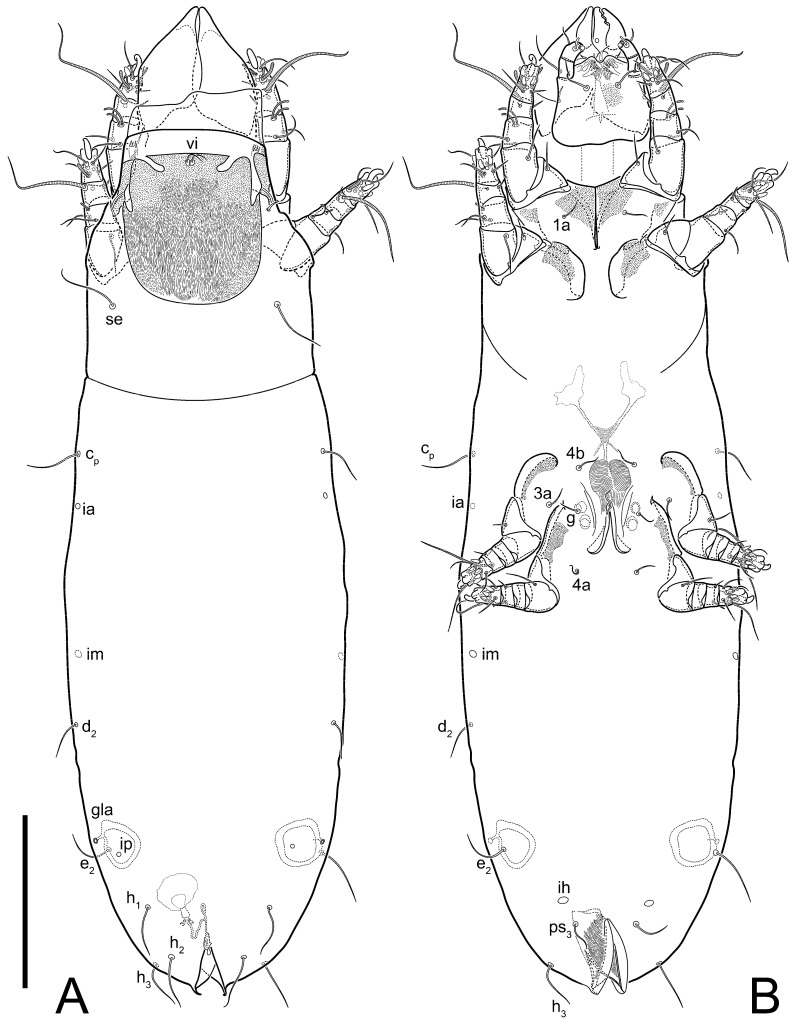
*Thyreophagus potawatomorum* sp. n., female (holotype): (**A**)—dorsal view; (**B**)—ventral view. Scale bar: 100 μm.

**Figure 22:**
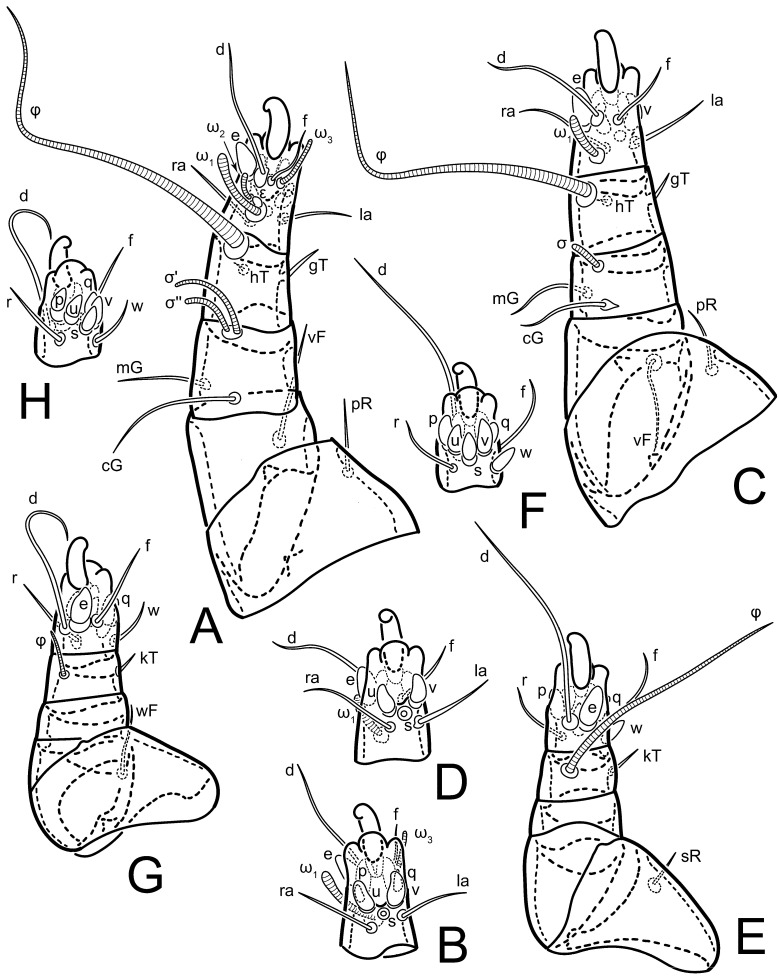
*Thyreophagus potawatomorum* sp. n., female (holotype): A—leg I, dorsal view; B—tarsus I, ventral view; C—leg II, dorsal view; D—tarsus II, ventral view; E—leg III, dorsal view; F—tarsus III, ventral view; G—leg IV, dorsal view; H—tarsus IV, ventral view. Scale bar: 50 μm.

**Figure 23:**
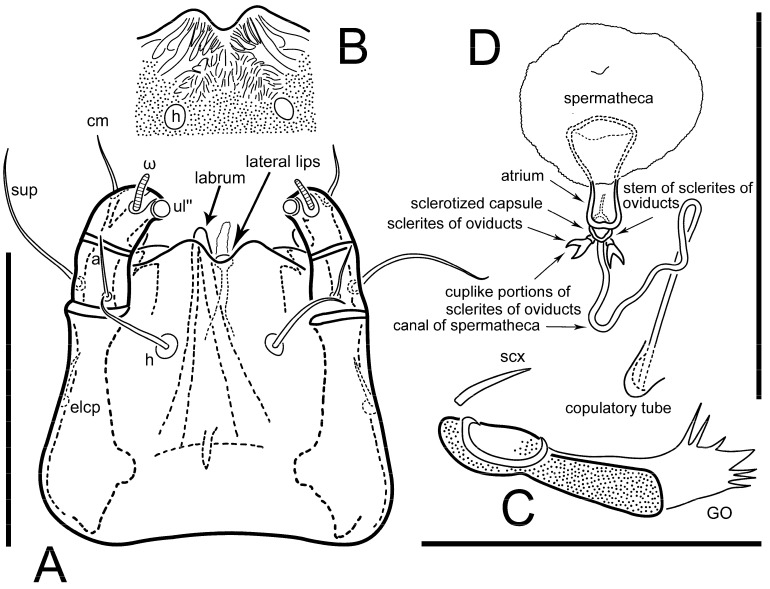
*Thyreophagus potawatomorum* sp. n., female (holotype): A—subcapitulum, ventral view; B—subcapitulum lobes, ventral view; C—supracoxal sclerite and Grandjean’s organ; D—spermatheca. Scale bar: 50 μm.

**Figure 24:**
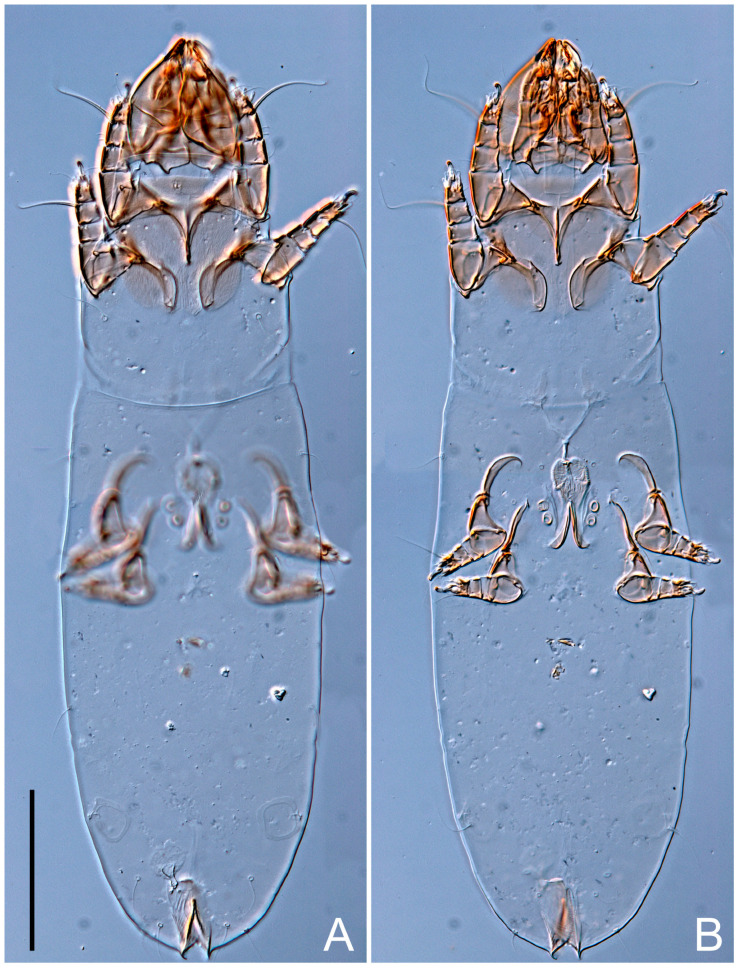
*Thyreophagus potawatomorum* sp. n., female (holotype), DIC images: (**A**)—dorsal view; (**B**)—ventral view. Scale bar: 100 μm.

**Figure 25:**
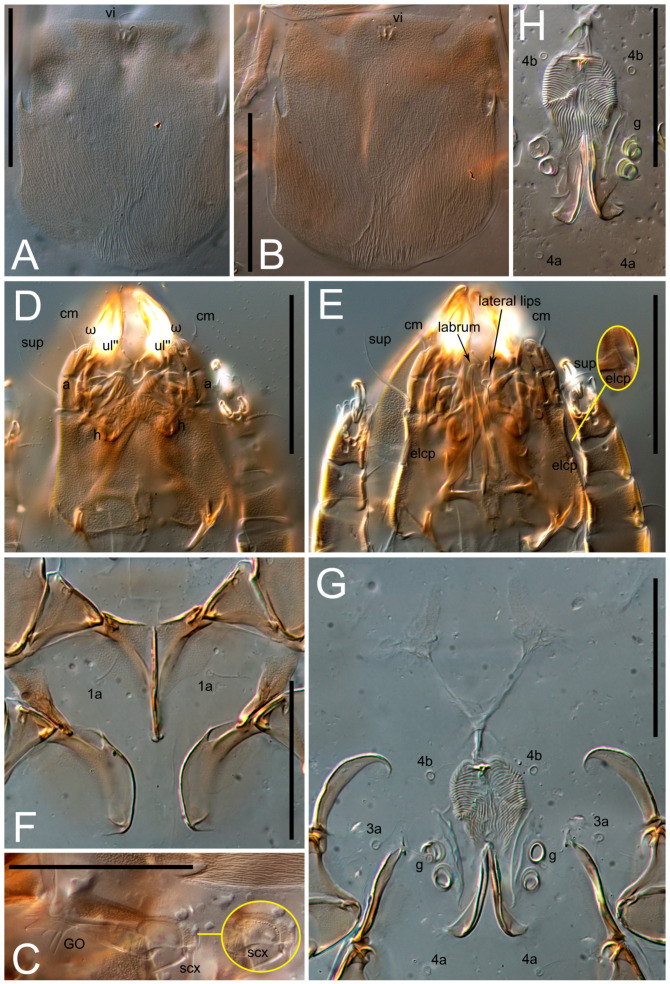
*Thyreophagus potawatomorum* sp. n., female ((**D**–**G**) holotype and (**A**–**C**,**H**) paratypes), DIC images: (**A**)—prodorsal shield; (**B**)—prodorsal shield; (**C**)—supracoxal sclerite and Grandjean’s organ; (**D**)—gnathosoma, ventral view; (**E**)—gnathosoma, optical section at level labrum, ventral view; (**F**)—coxal fields I–II; (**G**)—ovipore and coxal fields III–IV; (**H**)—ovipore. Scale bar: 50 μm.

**Figure 26:**
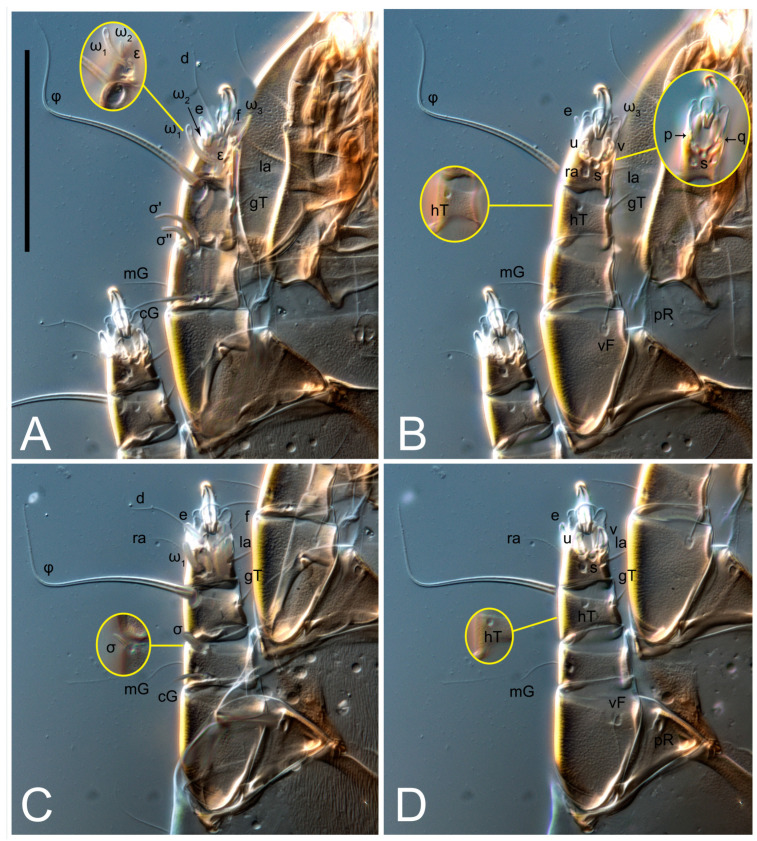
*Thyreophagus potawatomorum* sp. n., female (holotype), DIC images: (**A**)—leg I, dorsal view; (**B**)—leg I, ventral view; (**C**)—leg II, dorsal view; (**D**)—leg II, ventral view. Scale bar: 50 μm.

**Figure 27:**
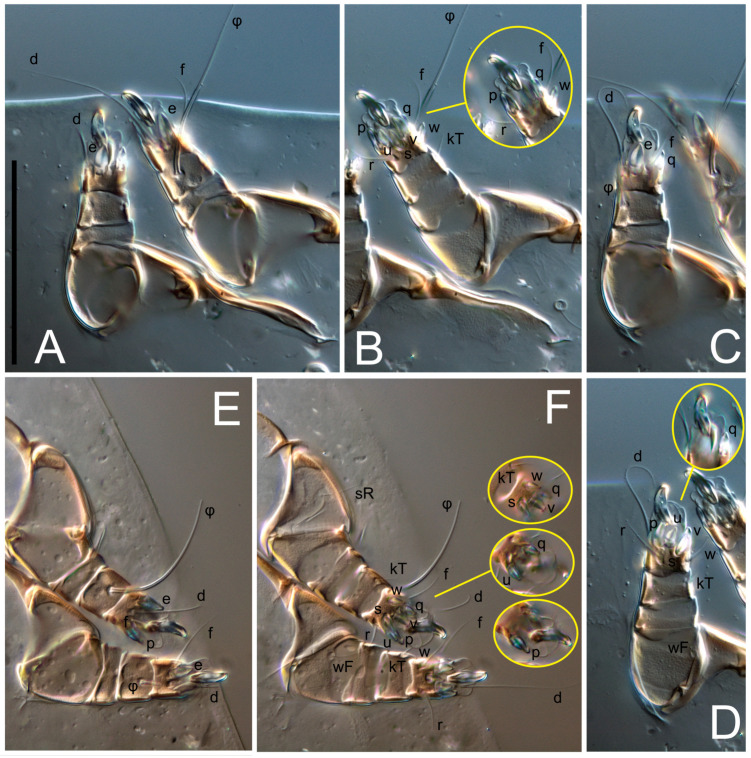
*Thyreophagus potawatomorum* sp. n., female ((**A**–**D**) holotype and (**E**,**F**) paratype), DIC images: (**A**)—legs III–IV, dorsal view; (**B**)—leg III, ventral view; (**C**)—leg IV, dorsal view; (**D**)—leg IV, ventral view; (**E**)—legs III–IV, dorsal view; (**F**)—legs III–IV, ventral view. Scale: bar 50 μm.

**Figure 28:**
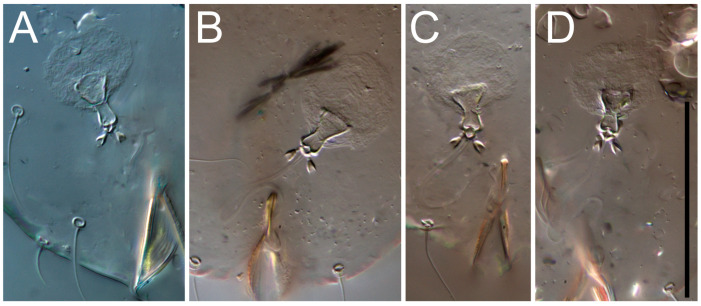
*Thyreophagus potawatomorum* sp. n., female ((**A**) holotype and (**B**–**D**) paratypes), spermatheca, DIC images. Scale bar: 50 μm.

**Female** (Figure 21, Figure 22, Figure 23, Figure 24, Figure 25, Figure 26, Figure 27 and Figure 28). Idiosoma elongate, 580 (holotype), 164 wide, 3.5-times longer than wide. Idiosomal cuticle smooth. Subcapitular setae (*h*) long, widened basally; palp tibial setae (*a*), lateral dorsal palp tibial setae (*sup*), dorsal palp tarsal seta (*cm*) filiform; supracoxal seta *elcp* present, widened basally; terminal palp tarsal solenidion ω short, bacilliform; external part of terminal eupathidium *ul*’’ dome-shaped; terminal eupathidium *ul*’ not observed. Prodorsal sclerite 88 (77–82, *n* = 6) long, 80 (65–70, *n* = 6) wide, 1.1 (1.1–1.2, *n* = 6)-times longer than wide, with setae *vi* (situated at anterior part of sclerite, bases separate but touching each other), rounded anterolateral incisions, and elongate midlateral incisions (insertion points of setae *ve*). Prodorsal sclerite punctate, with longitudinal linear pattern extending anteriorly from posterior end of sclerite and covering area exceeding 75% of sclerite; anterior lateral and medial areas have only punctate patterns; lines form a triangle at posterior end of sclerite. Grandjean’s organ (GO) with seven membranous finger-like processes; central process distinctly wider than remaining processes. Supracoxal seta (*scx*) smooth, sword-shaped, widened and flattened, tapering at tip, slightly curved. Idiosomal setae (*vi*, *se*, *c*_p_, *d*_2_, *e*_2_, *h*_1_, *h*_2_, *h*_3_, *ps*_3_) smooth, filiform, short and slender; opisthosomal gland openings slightly anteriad of setal bases *e*_2_. Four pairs of fundamental cupules (*ia*, *im*, *ip*, *ih*) present.

Ventral idiosoma with four pairs of coxal setae (*1a*, *3a*, *4a*, and *4b*) and one pair of genital setae (*g*). Shape of coxal sclerites as shown in Figure 1B and Figure 5F,G. Ovipore between coxal fields III and IV; genital valves shaped as an inverted Y; epigynal and medial apodemes well-developed. Genital papillae medium-sized, their diameter approximately 5–6-times shorter than length of genital setae. Anal opening on posterior margin of idiosoma, mostly ventral, with substantial portion positioned terminally and dorsally. Copulatory tube situated anteriad of dorsal end of anus. Canal of spermatheca long, slender tube of uniform width, not widened at entrance to spermatheca; atrium of spermatheca vase-shaped (length greater than width) with a rounded capsule at junction with canal of spermatheca. Small, paired Y-shaped sclerites of oviducts with short stems.

Legs short, all segments free. Trochanters I–III each with filiform seta, *pR* I–II, *sR* III; trochanter IV without setae. Femoral setation 1-1-0-1; setae *vF* I–II and *wF* IV long, filiform. Genual setation 2-2-0-0; setae *mG* and *cG* I–II long, filiform; seta *nG* III absent. Tibial setation 2-2-1-1; setae *hT* I-II small, short, thin; setae *gT* I–II elongated, somewhat spiniform; setae *kT* III–IV elongated, somewhat spiniform. Tarsal setation 10-10-10-10; all pretarsi with hooked empodial claws and short, paired condylophores. Tarsus I with 10 setae; *ra*, *la*, *f*, and *d* filiform; *e*, *u*, *v*, *p*, *q* spiniform; *s* flattened, button-shaped; setae *wa* absent. Tarsus II setation similar to that of tarsus I, except proral setae (*p*, *q*) represented by small triangular rudiments. Tarsus III with 10 setae; *f*, *d*, *r* filiform; *e*, *w*, *s*, *u*, *v*, *p*, *q* spiniform. Tarsus IV similar to tarsus III, except *w* filiform. Solenidion ω_1_ on tarsus I cylindrical, with clavate apex, bent and pointed outward, to posterior side of tarsus; solenidion ω_1_ on tarsus II simple, cylindrical, with clavate apex, not bent, shorter than ω_1_ on tarsus I. Solenidion ω_2_ on tarsus I shorter than ω_1_, cylindrical, with rounded apex, slightly widened at tip, situated slightly anterior and external to ω_1_. Solenidion ω_3_ on tarsus I cylindrical, with rounded apex, shorter than ω_1_, longer than ω_2_. Famulus (ε) of tarsus I wide, spiniform, with broadly rounded apex. Solenidia φ of tibiae I–III elongate, tapering, well extending beyond apices of respective tarsi with ambulacra; solenidion φ IV shorter than tarsus IV (with ambulacra). On genu I, solenidion σ’ elongate, with rounded tip, slightly not reaching bases of φ I, about 1.5-times longer than σ’’; σ’’ I distinctly wider than σ’, about half the length of tibia I. On genu II, solenidion σ short (more than three-times longer than its width), with rounded tip. Solenidia σ III and IV absent.

**Male**. Absent.

**Figure 29:**
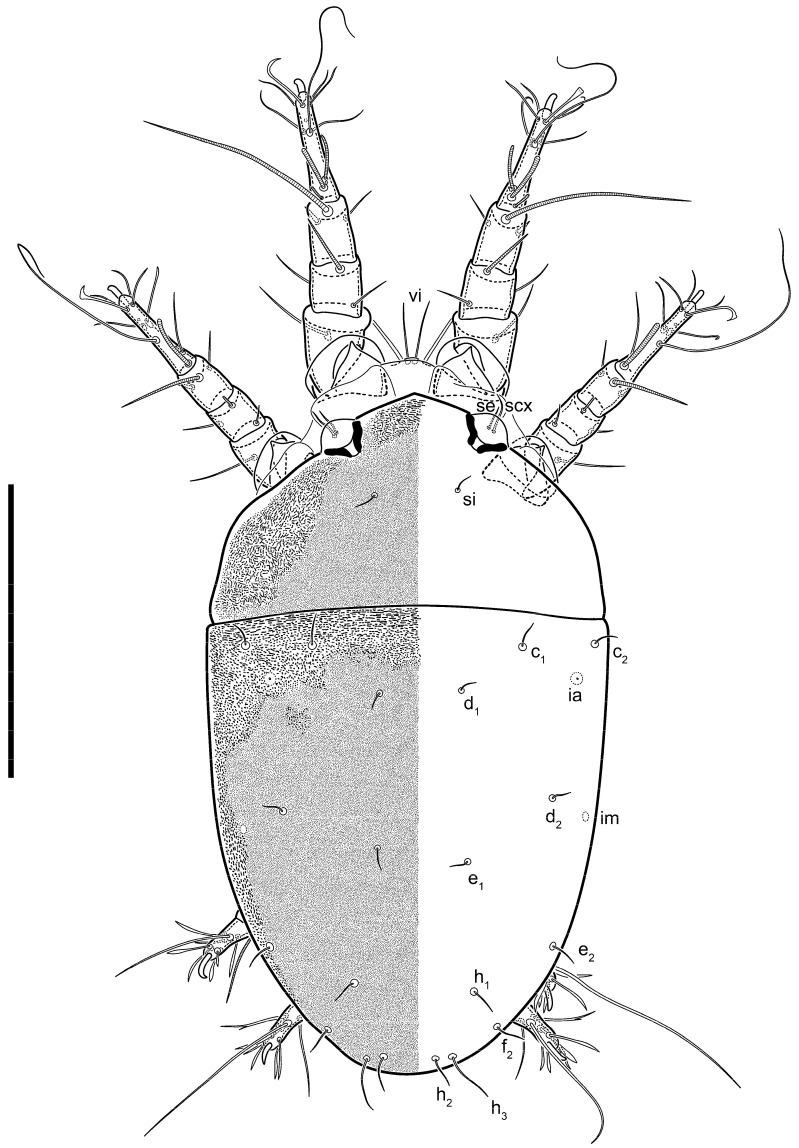
*Thyreophagus potawatomorum* sp. n., heteromorphic deutonymph (paratype), dorsal view. Scale bar: 100 μm.

**Figure 30:**
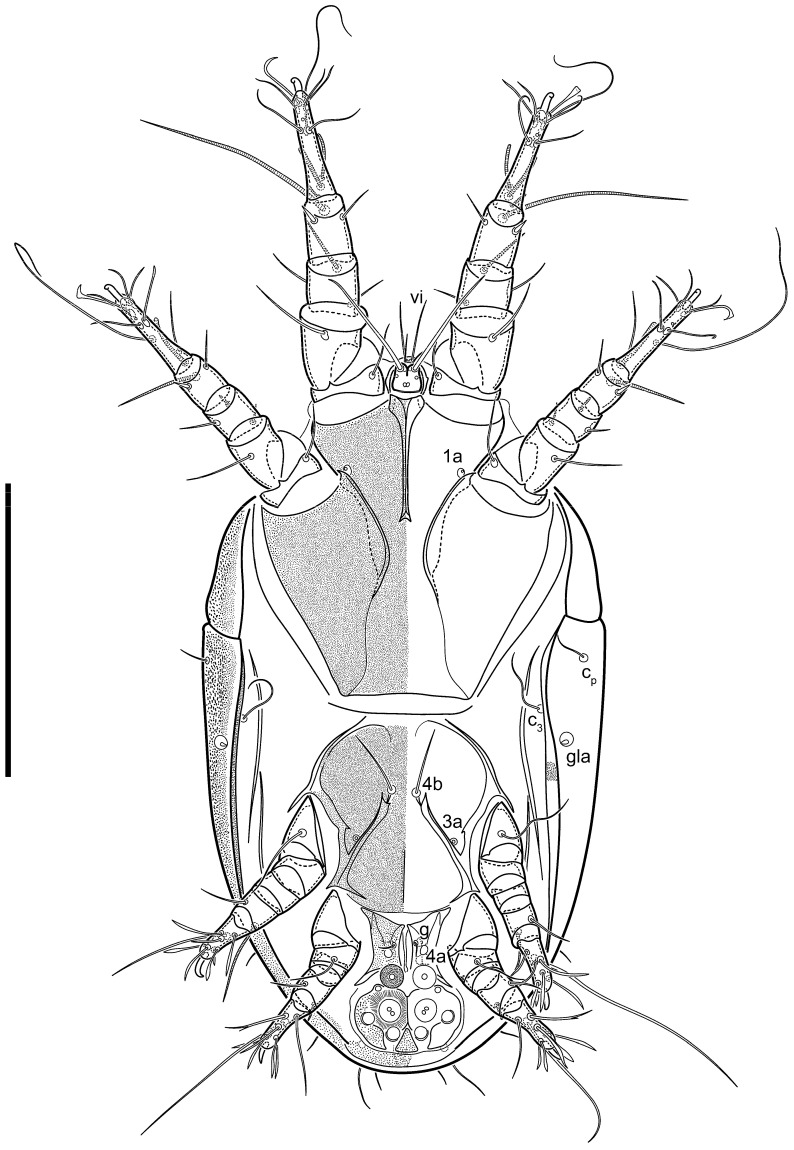
*Thyreophagus potawatomorum* sp. n., heteromorphic deutonymph (paratype), ventral view. Scale bar: 100 μm.

**Figure 31:**
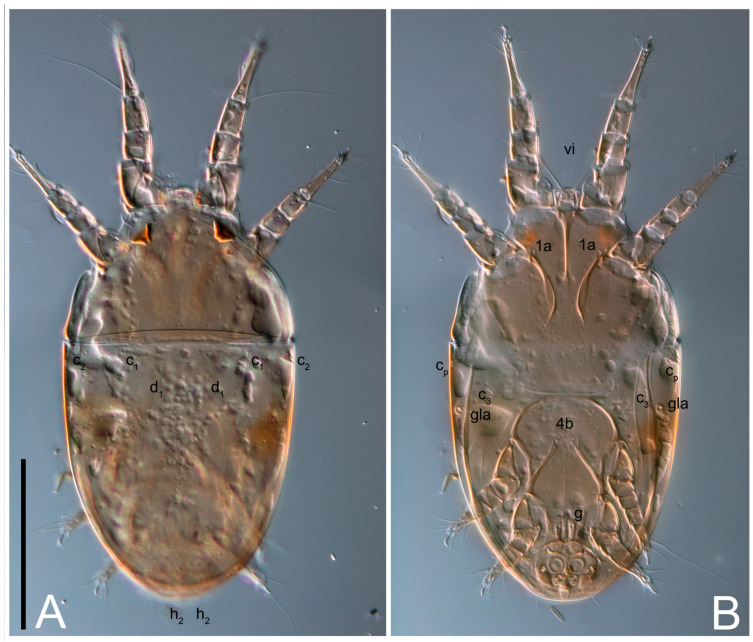
*Thyreophagus potawatomorum* sp. n., heteromorphic deutonymph (paratype), DIC images: (**A**)—dorsal view; (**B**)—ventral view. Scale bar: 100 μm.

**Figure 32:**
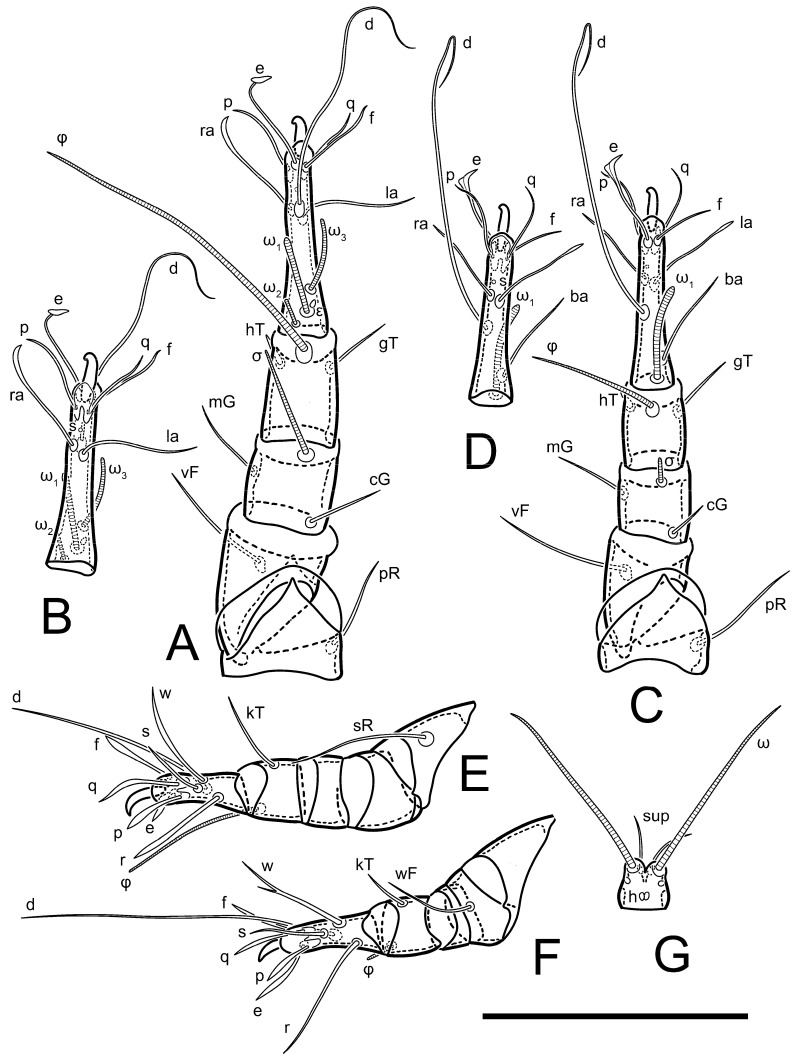
*Thyreophagus potawatomorum* sp. n., heteromorphic deutonymph (paratype): A—leg I, dorsal view; B—tarsus I, ventral view; C—leg II, dorsal view; D—tarsus II, ventral view; E—leg III, ventral view; F—leg IV, ventral view; G—gnathosoma, ventral view. Scale bar: 50 μm.

**Figure 33:**
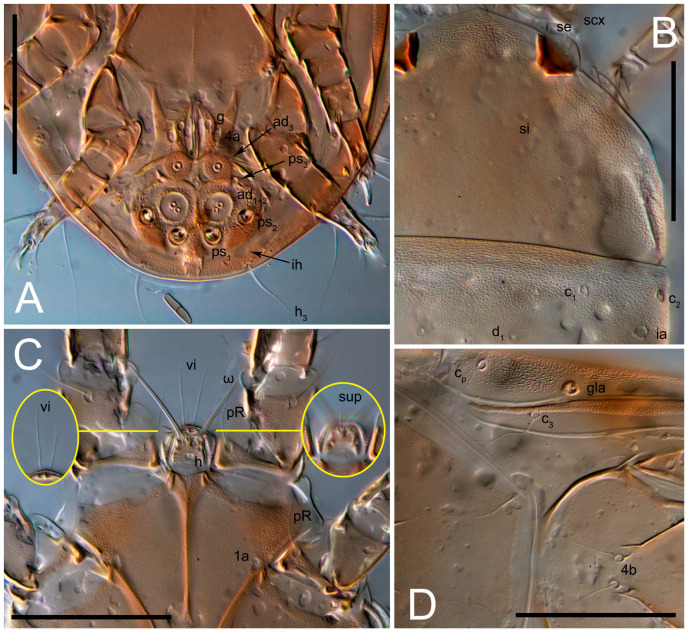
*Thyreophagus potawatomorum* sp. n., heteromorphic deutonymph (paratype), DIC images: (**A**)—hysterosoma, part, ventral view; (**B**)—propodosoma and hysterosoma, part, dorsal view; (**C**)—gnathosoma and propodosoma, part, ventral view; (**D**)—propodosoma and hysterosoma, part, ventral view. Scale bar: 50 μm.

**Figure 34:**
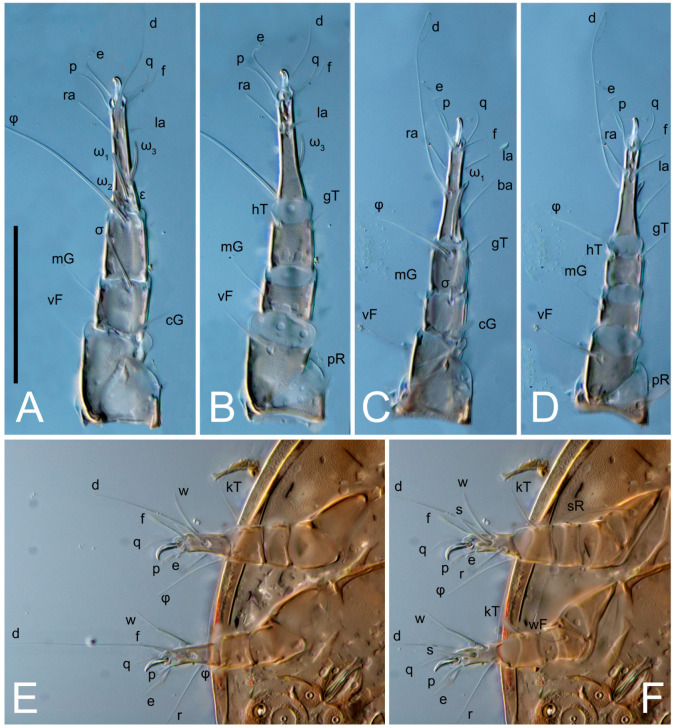
*Thyreophagus potawatomorum* sp. n., heteromorphic deutonymph (paratype), DIC images: (**A**)—leg I, dorsal view; (**B**)—leg I, ventral view; (**C**)—leg II, dorsal view; (**D**)—leg II, ventral view; (**E**)—legs III–IV, dorsal view; (**F**)—legs III–IV, ventral views. Scale bar: 50 μm.

**Heteromorphic deutonymph** (*n* = 1) (Figure 29, Figure 30, Figure 31, Figure 32, Figure 33 and Figure 34). Body elongate, 1.67-times longer than wide, widest in sejugal region; idiosomal length 235, width 140. Gnathosoma short, subcapitulum and palp fused, with apical palpal solenidia ω and filiform apicodorsal setae *(sup*); setae *h* absent from subcapitular remnant, their positions marked by somewhat refractile spots.

Dorsum. Propodosomal and hysterosomal smoothly punctate; distinct linear pattern present on anterior and lateral sides of propodosomal sclerite and hysterosomal shield. Apex of propodosomal sclerite shaped as obtuse triangle. Internal vertical setae (*vi*), sitiated on apex of propodosoma, long, bases separated. A pair of lateral ocelli present on propodosoma; widely separated from each other (distance 36, *n* = 1); lenses and pigmented spots present, maximal diameter of lenses 16. External vertical setae (*ve*) absent; external scapular setae *se* situated just below eye lenses; internal scapular setae (*si*) distinctly posterior and medial to external scapulars (*se*). Supracoxal setae of legs I (*scx*) filiform, situated below *se*. Sejugal furrow well-developed. Propodosomal sclerite 73, hysterosomal shield 160, ratio hysterosoma shield/propodosomal sclerite, length = 2.19. Hysterosoma with 11 pairs of simple, filiform setae on hysterosomal shield (*c*_1_, *c*_2_, *c*_p_, *d*_1_, *d*_2_, *e*_1_, *e*_2_, *f*_2_, *h*_1_, *h*_2_, *h*_3_), setae *h*_3_ distinctly longer than others. Opisthonotal gland openings (*gla*) ventral; situated slightly posterior to setae *c_3_* off hysterosomal shield. Of four fundamental pairs of cupules, only three pairs observed: *ia* posteriomedial of setae *c*_2_, *im* posterior of *d*_2_ level and *ih* ventral, lateral to posterior sides of attachment organ.

Venter. Coxal fields sclerotized, smoothly punctate. Anterior apodemes of coxal fields I fused forming sternum. Sternum not reaching posterior border of sternal shield by distance exceeding its length. Posterior border of sternal shield weakly sclerotized. Anterior apodemes of coxal fields II curved medially. Posterior apodemes of coxal fields II weakly developed, thin. Sternal and ventral shields adjacent. Anterior apodemes of coxal fields III free. Posterior medial apodeme present between coxal fields IV, well-separated from anterior apodemes IV and genital opening. Posterior apodemes IV absent. Dorsal hysterosomal shield separated from ventral surface by a distinct suture on each side. Subhumeral setae (*c*_3_) long, filiform, positioned on ventral surface between legs II–III, adjacent to region separating sternal and ventral shields. Coxal setae *1a*, *3a* reduced, represented by minute structures each situated in an alveolus. Setae *4b*, *g* filiform; *4a* in form of small, rounded conoids, *4b* longer than *g*. Genital region in posterior portion of coxal fields IV; genital opening elongate, there are two pairs of genital papillae inside progenital atrium; papillae two-segmented, with rounded apices. Coxal setae (*4b*) situated at tips anterior to coxal apodemes IV; genital setae (*g*) situated laterad of genital opening. Attachment organ posterior to coxal fields IV. Anterior suckers (*ad*_3_) round, median suckers (*ad*_1+2_) distinctly larger, with paired vestigial alveoli; pair of small refractile spots anterolateral to median suckers (*ps*_3_); lateral conoidal setae of attachment organ (*ps_2_*) situated slightly posterior to a line joining centers of median suckers, distinctly anterior conoidal setae (*ps*_1_) and slightly posterior to median suckers (*ad*_1+2_); anterior and posterior lateral and posterior median cuticular conoids well-developed; anus positioned between anterior suckers (*ad*_3_).

Legs. Legs elongate, all segments free. Trochanters I–III each with long, filiform seta, *pR* I–II, *sR* III. Femoral setation 1-1-0-1; setae *vF* I–II and *wF* IV long, filiform. Genual setation 2-2-0-0; setae *mG* and *cG* I–II filiform, seta *nG* III absent. Tibial setation 2-2-1-1; setae *hT* I and II spiniform; setae *gT* I filiform; setae *kT* III filiform; *kT* IV somewhat spiniform. Tarsal setation 7-8-8-8. All pretarsi consisting of hooked empodial claws arising from tarsal apices, attached to short paired condylophores within tarsal apices. Tarsus I with three filiform setae (*p*, *q* and *d*), three slightly foliate seate (*ra*, *la* and *f*), and one spoon-shaped seta *e*. Seta *d* I elongate, longer than tarsus (its base situated at level of setae *ra* and *la*); seta *s* I alveolar; setae *wa*, *aa* and *ba* I absent. Tarsus II similar to tarsus I except seta *ba* present and filiform, base of seta *d* posterior to level of setae *ra* and *la*. Tarsus III with eight setae (*w*, *r*, *s*, *p*, *q*, *e*, *f*, *d*) smooth, all setae, except *d* III foliate. Tarsus IV similar to tarsus III, except seta *r* longer, filiform, and setae *w* filiform and has a distinct prong. Solenidia ω_1_ on tarsi I–II cylindrical, with slightly clavate apices, ω_1_ II longer than ω_1_ I. Solenidion ω_3_ on tarsus I slightly shorter than ω_1_, with rounded apex, positioned slightly anterior to ω_1_; famulus (ε) bulbous, situated between ω_1_ and ω_3_. Solenidion ω_2_ of tarsus I thin, with rounded apex, positioned somewhat more basal and posterior to ω_1_+ε+ ω_3_ group. Solenidia φ of tibiae I–III elongate, tapering; φ I and III, longer than tarsus I and III, respectively; φ II shorter than tarsus II; φ IV short. Solenidion σ of genu I elongate, slightly tapering, nearly reaching tip of tibia I; σ of genu II shorter, cylindrical, not reaching midlength of tibia II; σ of genu III absent.

**Diagnosis**

Females. *Thyreophagus potawatomorum* is similar to *Th. spinitarsis* (Fain, 1982) by the linear striations of the prodorsal sclerite extending over at least 75% of the sclerite length and by tarsi III having seven spiniform setae (*e*, *u*, *v*, *p*, *q*, *s*, *w*), but differs by the widened, vase-shaped atrium (dome-shaped in *Th. spinitarsis*). *Th. potawatomorum* is very close to *Th. berxi* sp. n., but differs by the following character states: setae *vi* are not extending beyond the anterior margin of the prodorsum (extending in *Th. berxi*); bases of *vi* touch each other (distinctly separated in *Th. berxi*), lines of the prodorsal sclerite are longer than the diameter of the bases of *vi* (shorter in *Th. berxi*); on the prodorsal sclerite, the anterior medial striated area differs from the posterior medial striated area (medial striated area has a uniform pattern in *Th. berxi*); opisthosomal setae *h*_2_ and *h*_3_ are shorter than the anus (distinctly longer in *Th. berxi*); a rounded capsule is present between the canal of spermatheca and the atrium (absent in *Th. berxi*).

Heteromorphic deutonymph. *Th. potawatomorum* is close to *Th. corticalis* and *Th. berxi* by the following character states: body elongate (more than 1.7-times longer than wide), setae *hT* and *gT* I–II are present, different in shape, bases of setae *d* are at the level with bases of setae of *ra* and *la* on tarsus I. *Th. potawatomorum* differs from *Th. corticalis* and *Th. berxi* by the following character states: posterior median apodeme present, well-developed (absent in *Th. corticalis* and weakly developed in *Th. berxi*); the diameter of ocelli is about 16 (19 in *Th. corticalis*; 23 in *Th. berxi*); the distance between the ocelli is 36 (60 in *Th. corticalis*; 50 in *Th. berxi* or); setae *wa* I are absent (present in *Th. corticalis*; absent in *Th. berxi*); on tibia IV, seta *kT* IV without distinct prong (with a distinct prong in *Th. berxi*; without in *Th. corticalis*).

*Thyreophagus berxi* Klimov, Kolesnikov, Wäckers, Merckx, Duarte, Vangansbeke, sp. n.

(Figure 35, Figure 36, Figure 37, Figure 38, Figure 39, Figure 40, Figure 41 and Figure 42).

urn:lsid:zoobank.org:pub:7C7FEAA7-EB57-4A12-A489-196D2BEEA5D9.

**Type material**. Holotype female, five paratype females—BELGIUM: East Flanders, Gentbrugge, Moscou, *Fagus sylvatica* (Fagales: Fagaceae) twig, subcortical, 51°01′50.7″ N 3°44′54.6″ E, 16 November 2019, Dominiek Vangansbeke, PBK 22-0905-033#slide1; one paratype female, same data, slide 2. One paratype female—BELGIUM: Flemish Brabant, Arenberg Castle, Leuven, *Fagus sylvatica* (label on slide = “19-1-20 Tr. Sp. LA Arenberg beech 1”), 50°51′32.6″ N 4°39′54.4″ E, 19 January 2020, Jonas Merckx, PBK 22-0905-034#slide1. Six paratype females, one paratype HDN—GERMANY: Saxony, Dresden, *Betula* (Fagales: Betulaceae) (original label says: “birch twig, Felix white bag” comment = most likely birch twig”), 51°05’38.7″ N 13°50’35.6″ E, 22 November 2020, Felix Wäckers (received via Dominiek Vangansbeke), PBK 22-0905-035#slide1. Six paratype females, three paratype HDNs—FRANCE: Grand Est, Verzenay, Marcus Duarte, 8 April 2023, DV_2023-037.

**Depository**. Holotype, paratypes (Belgium, Germany)—University of Michigan, Museum of Zoology, Ann Arbor, Michigan, USA. Paratypes (France)—Royal Belgian Institute of Natural Sciences, Brussels, Belgium.

**Etymology**. The new species is named after the Belgian entomologist Peter Berx.

**Habitat***. Thyreophagus berxi* lives under the bark of small fallen branches of deciduous trees, such as European beech and birch trees.

**Description**

**Figure 35:**
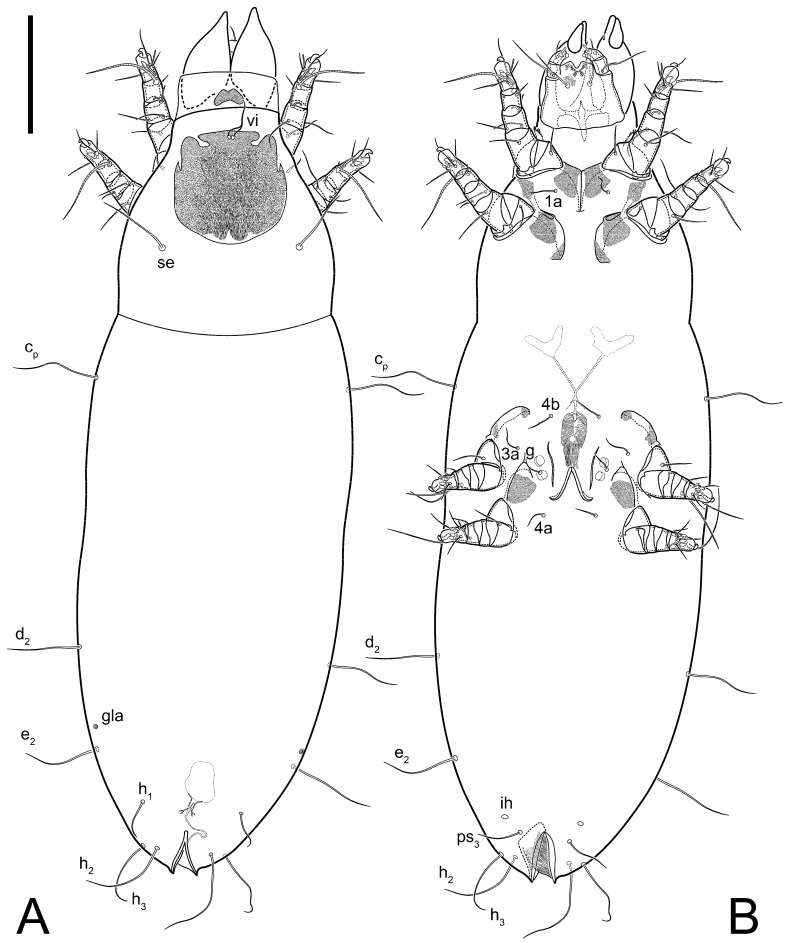
*Thyreophagus berxi* sp. n., female (holotype): (**A**)—dorsal view; (**B**)—ventral view. Scale bar: 100 μm.

**Figure 36:**
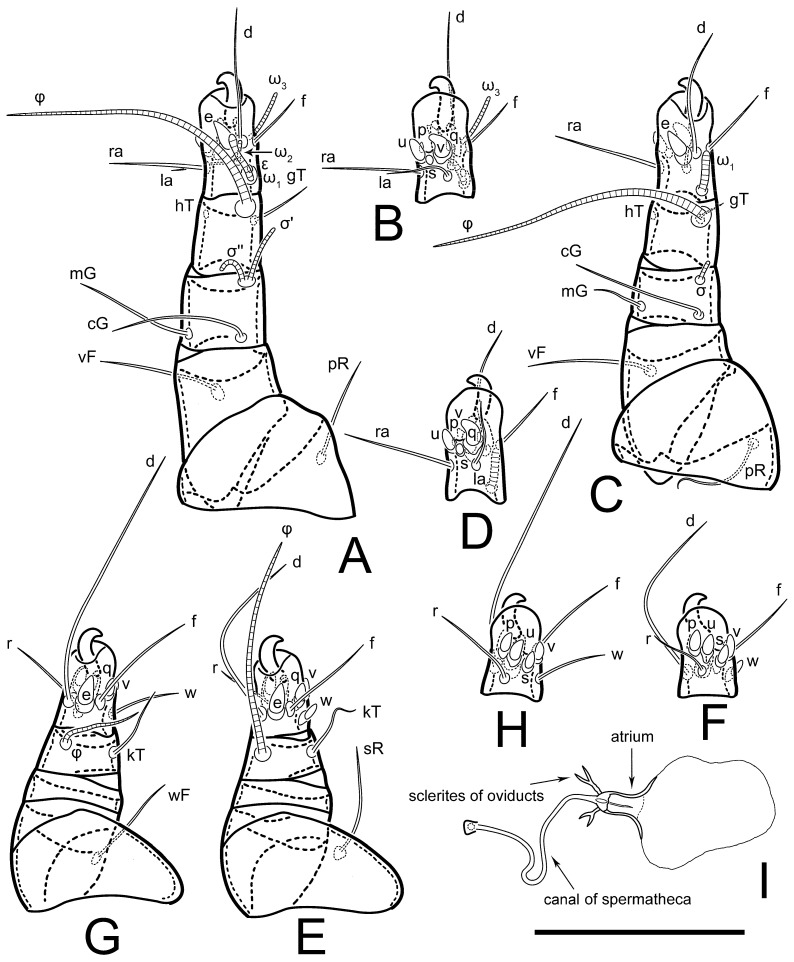
*Thyreophagus berxi* sp. n., female (holotype): A—leg I, dorsal view; B—tarsus I, ventral view; C—leg II, dorsal view; D—tarsus II, ventral view; E—leg III, dorsal view; F—tarsus III, ventral view; G—leg IV, dorsal view; H—tarsus IV, ventral view; I—spermatheca. Scale bar: 50 μm.

**Figure 37:**
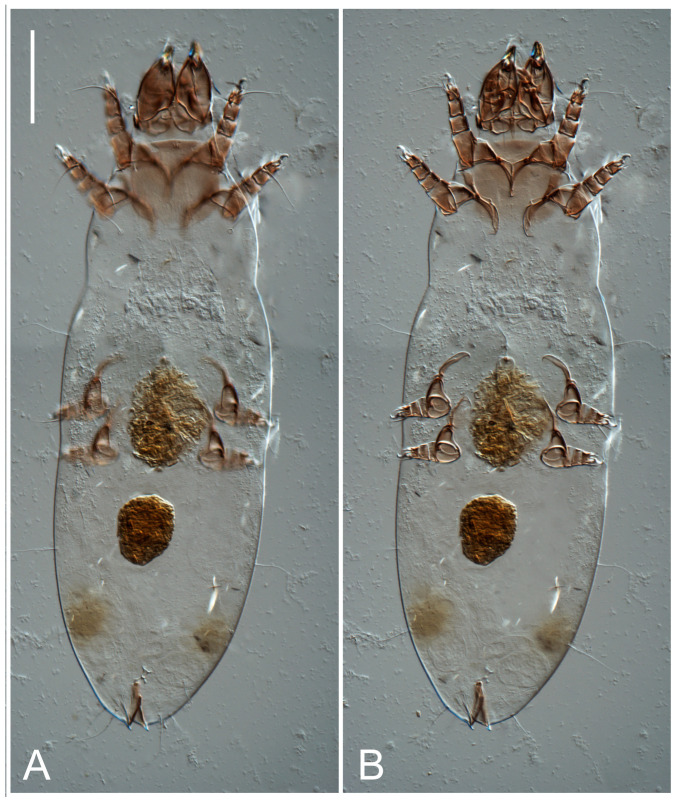
*Thyreophagus berxi* sp. n., female (holotype), DIC images: (**A**)—dorsal view; (**B**)—ventral view. Scale bar: 100 μm.

**Figure 38:**
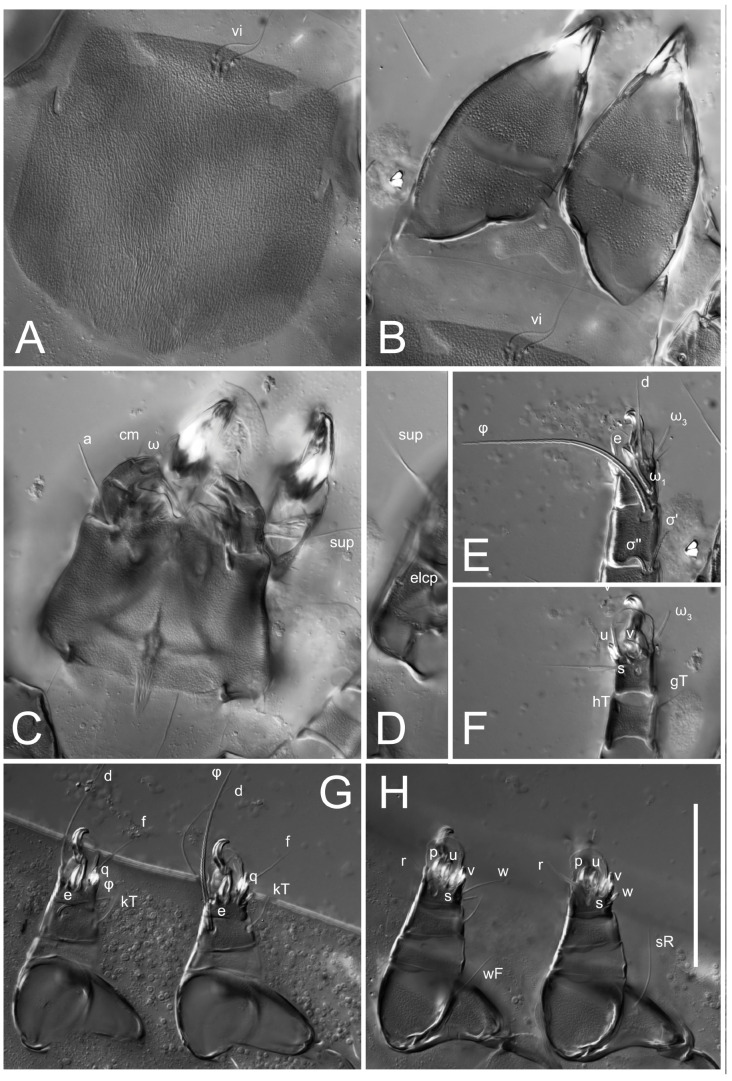
*Thyreophagus berxi* sp. n., female (holotype), DIC images: (**A**)—prodorsal shield; (**B**)—gnathosoma, dorsal view; (**C**)—gnathosoma, ventral view; (**D**)—lateral part of subcapitulum with seta *elcp*; (**E**)—tibia and tarsus I, dorsal view; (**F**)—tibia and tarsus I, ventral view; (**G**)—legs III and IV, dorsal view; (**H**)—legs III and IV, ventral view. Scale bar: 50 μm.

**Figure 39:**
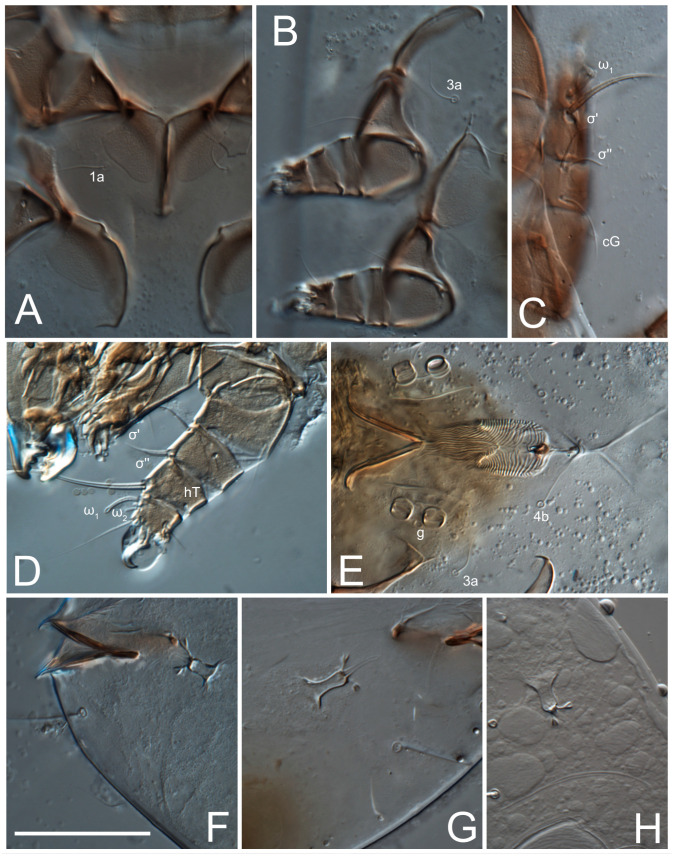
*Thyreophagus berxi* sp. n., female ((**A**,**B**,**E**,**F**) holotype and (**C**,**D**,**G**,**H**) paratypes), DIC images: (**A**)—coxal fields I–II; (**B**)—coxal fields III–IV; (**C**)—leg I, dorsal view; (**D**)—leg I, antiaxial view; (**E**)—ovipore; (**F**–**H**)—spermatheca. Scale bar: 50 μm.

**Female** (Figure 35, Figure 36, Figure 37, Figure 38 and Figure 39). Idiosoma elongate, 670 (600–630, *n* = 2) (holotype, range), 230 (200–240, *n* = 2) wide, 2.9 (2.5–3.1, *n* = 2)-times longer than wide. Idiosomal cuticle smooth. Subcapitular setae (*h*) long, widened basally; palp tibial setae (*a*), lateral dorsal palp tibial setae (*sup*), dorsal palp tarsal seta (*cm*) filiform; supracoxal seta *elcp* present, widened basally; terminal palp tarsal solenidion ω short, bacilliform; external part of terminal eupathidium *ul*’’ dome-shaped; terminal eupathidium *ul*’ small, dome-shaped. Prodorsal sclerite 98 (100) long, 94 (96) wide, 1.1 (1.1, *n* = 1)-times longer than wide, with setae *vi* (situated at anterior part of shield, bases distinctly separated), rounded anterolateral incisions, and elongate midlateral incisions (insertion points of setae *ve*). Prodorsal sclerite has three types of patterns: smoothly punctated (anterior lateral 1/3 and anterior medial 1/2 parts of sclerite), longitudinal lines (posterior medial part of sclerite), smoothly punctated smaller lines (posterior lateral part). Grandjean’s organ (GO) with 9–10 membranous finger-like extensions. Supracoxal seta (*scx*) smooth, sword-shaped, widened and flattened, tapering at tip, slightly curved. Idiosomal setae (*vi*, *se*, *c*_p_, *d*_2_, *e*_2_, *h*_1_, *h*_2_, *h*_3_, *ps*_3_) smooth, filiform, medium length and slender; opisthosomal gland openings slightly anteriad of setal bases *e*_2_. Only one pair of fundamental cupules (*ih*) observed.

Ventral surface of idiosoma with four pairs of coxal setae (*1a*, *3a*, *4a*, and *4b*) and one pair of genital setae (*g*). Shape of coxal sclerites as in Figure 1B and Figure 5A,B. Ovipore between coxal fields III and IV; genital valves shaped as an inverted Y; epigynal and medial apodeme well-developed. Genital papillae medium-sized, their diameter approximately 0.3–0.4-times the length of coxal and genital setae. Anal opening on posterior margin of idiosoma, mostly ventral, with substantial portion situated terminally and dorsally. Copulatory tube present, short, situated anteriad of dorsal end of anus, with opening developed. Canal of spermatheca long, slender, uniformly wide, except slightly widened at entrance to atrium of spermatheca. Atrium vase-shaped, length greater than width. A capsule between atrium and canal of spermatheca absent. Y-shaped sclerites of oviducts with long stems.

Legs short, all segments free. Trochanters I–III each with filiform seta, *pR* I–II, *sR* III; trochanter IV without setae. Femoral setation 1-1-0-1; setae *vF* I–II and *wF* IV long, filiform. Genual setation 2-2-0-0; setae *mG* and *cG* I–II long, filiform; seta *nG* III absent. Tibial setation 2-2-1-1; setae *hT* I–II alveoli or small, short, and thin; setae *gT* I–II elongated, somewhat spiniform; setae *kT* III–IV elongated, somewhat spiniform. Tarsal setation 10-10-10-10; all pretarsi with hooked empodial claws and short paired condylophores. Tarsus I and II with 10 setae; *ra*, *la*, *f*, and *d* filiform; *e*, *u*, *v*, spiniform; *s* flattened, button-shaped; proral setae (*p*, *q*) represented by small triangular rudiments; setae *wa* absent. Tarsus III with 10 setae; *f*, *d*, *r* filiform; *e*, *w*, *s*, *u*, *v*, *p*, *q* spiniform. Tarsus IV similar to tarsus III, except *w* filiform. Solenidion ω_1_ on tarsus I cylindrical, with clavate apex, bent and pointed outward, to posterior side of tarsus; solenidion ω_1_ on tarsus II simple, cylindrical, with clavate apex, not bent, shorter than ω_1_ on tarsus I. Solenidion ω_2_ on tarsus I shorter than ω_1_, cylindrical, with rounded apex, slightly widened at tip, situated slightly anterior and external to ω_1_. Solenidion ω_3_ on tarsus I cylindrical, with rounded apex, as long as ω_1_, longer than ω_2_. Famulus (ε) of tarsus I wide, spiniform, with broadly rounded apex. Solenidia φ of tibiae I–III elongate, tapering, well extending beyond apices of respective tarsi with ambulacra; solenidion φ IV shorter, shorter than tarsus IV (with ambulacra). On genu I, solenidion σ’ elongate, with rounded tip, reaching bases of φ I; σ’’ I distinctly wider than σ’, slightly not reaching bases of φ I. On genu II, solenidion σ short (more than three-times longer than its width), with rounded tip. Solenidion σ of genu III and IV absent.

**Male**. Absent.

**Figure 40:**
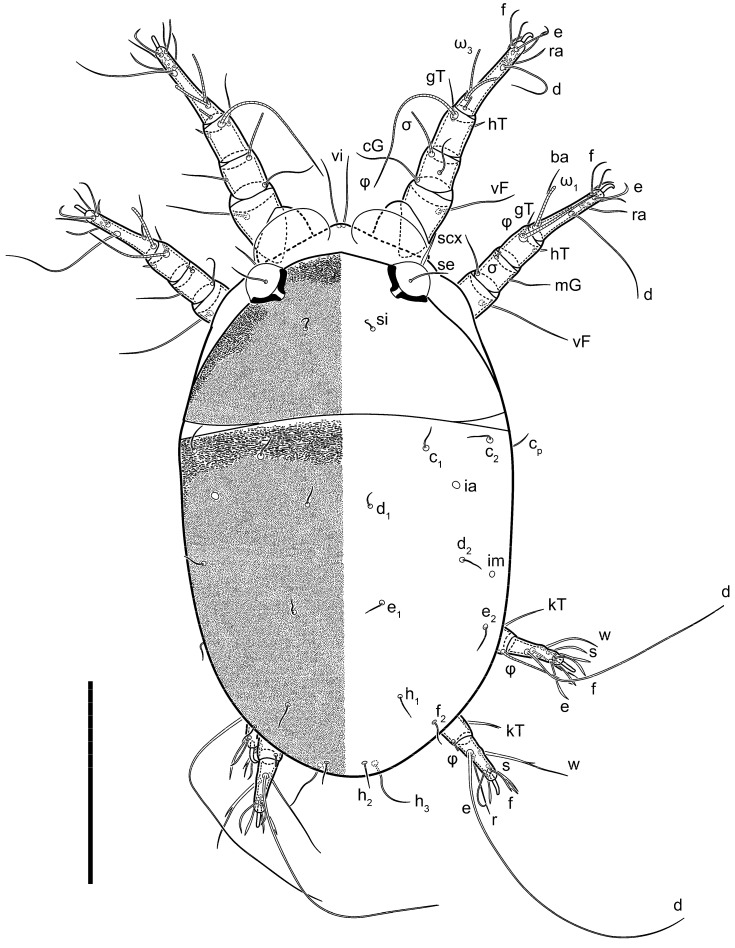
*Thyreophagus berxi* sp. n., heteromorphic deutonymph (paratype), dorsal view. Scale bar: 100 μm.

**Figure 41:**
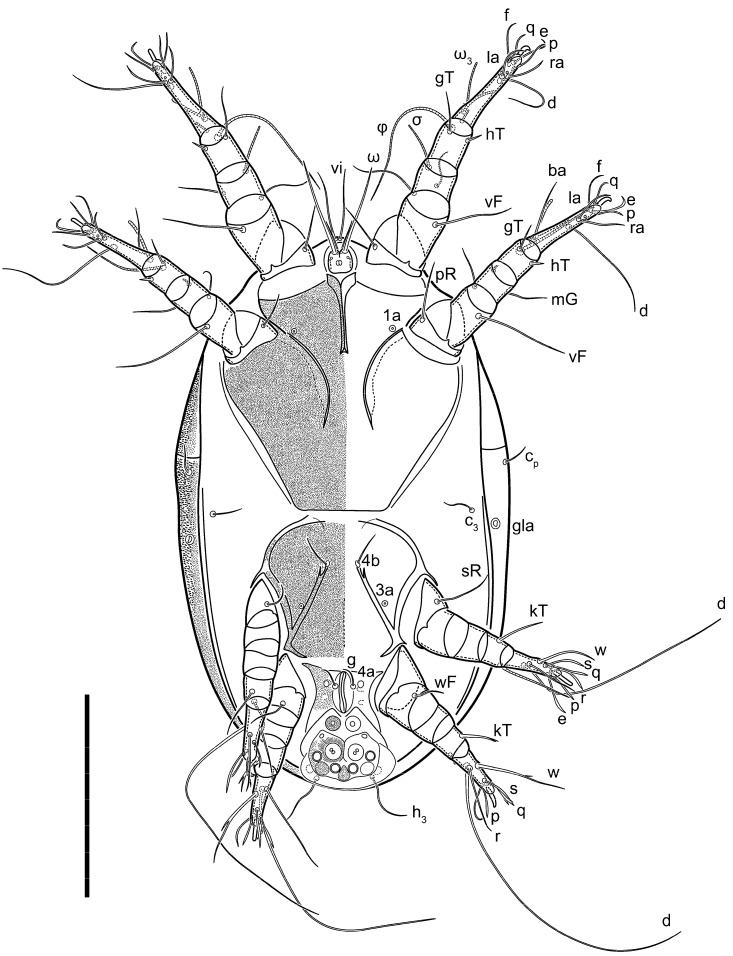
*Thyreophagus berxi* sp. n., heteromorphic deutonymph (paratype), ventral view. Scale bar: 100 μm.

**Figure 42:**
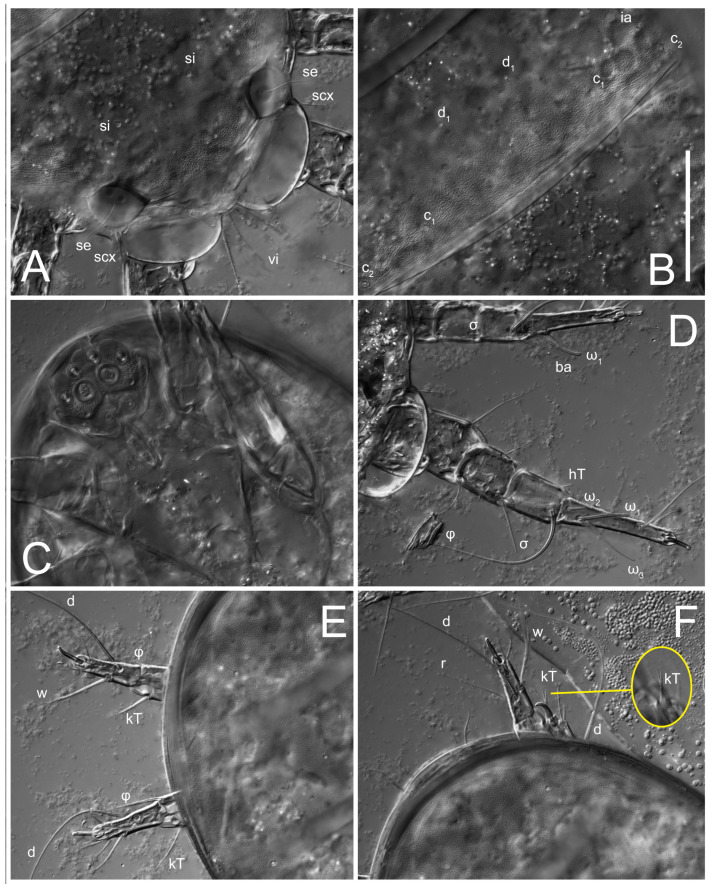
*Thyreophagus berxi* sp. n., heteromorphic deutonymph (paratype), DIC images: (**A**)—propodosoma, dorsal view; (**B**)—sejugal furrow; (**C**)—hysterosoma, part, ventral view; (**D**)—legs I and II, dorsal view; (**E**,**F**)—legs III and IV and hysterosoma, part, dorsal view. Scale bar: 50 μm.

**Heteromorphic deutonymph** (*n* = 1) (Figure 40, Figure 41 and Figure 42). Body elongate, 1.7-times longer than wide, widest in sejugal region; idiosomal length 270, width 160. Gnathosoma short, subcapitulum and palp fused, with apical palpal solenidia (ω) and filiform apicodorsal setae (*sup*); setae *h* absent from subcapitular remnant, their positions marked by somewhat refractile spots.

Dorsum. Propodosomal sclerite and hysterosomal shield smoothly punctate; distinct linear pattern present on anterior and lateral sides of propodosomal sclerite. A small area of linear pattern present on hysterosomal shield. Anterior end p of propodosoma shaped as obtuse triangle. Internal vertical setae (*vi*) apical, long, bases separated. A pair of lateral ocelli present on propodosoma; ocelli widely separated from each other (distance 50); lenses and pigmented spots present, maximum diameter of lenses 23. External vertical setae (*ve*) absent; external scapular setae *se* situated just below eye lenses; internal scapular setae (*si*) distinctly posterior and medial to external scapulars (*se*). Supracoxal setae of legs I (*scx*) filiform, situated below setae *se*. Sejugal furrow well-developed. Propodosomal sclerite 80, hysterosomal shield 180, ratio hysterosomal shield/propodosomal sclerite length = 2.25. Hysterosomal shield with 11 pairs of simple, filiform setae (*c*_1_, *c*_2_, *c*_p_, *d*_1_, *d*_2_, *e*_1_, *e*_2_, *f*_2_, *h*_1_, *h*_2_, *h*_3_), setae *h*_3_ distinctly longer than others. Opisthonotal gland openings (*gla*) ventral; situated ventrally on hysterosomal shield, slightly posterior to setae *c*_3_. Of four fundamental pairs of cupules, only three pairs observed: *ia* posteriomedial of setae *c*_2_, *im* posterior to level of *d*_2_ and *ih* ventral, lateral to posterior sides of attachment organ.

Venter. Coxal fields sclerotized, smoothly punctate. Anterior apodemes of coxal fields I fused forming sternum. Sternum not reaching posterior border of sternal shield by distance exceeding its length. Posterior border of sternal shield weakly sclerotized. Anterior apodemes of coxal fields II curved medially. Posterior apodemes of coxal fields II weakly developed, thin, sternal and ventral shield adjacent. Anterior apodemes of coxal fields III free. Posterior medial apodeme in area of coxal fields IV weakly expressed. Posterior apodemes IV absent. Subhumeral setae (*c*_3_) long, filiform, situated ventrally between legs II–III, adjacent to region separating sternal and ventral shields. Coxal setae *1a*, *3a* reduced, represented by minute structures each situated in an alveolus. Setae *4b*, *g* filiform; *4a* small, rounded conoids, *4b* longer than *g*. Genital region in posterior portion of coxal fields IV; genital opening elongate; there are two pairs of genital papillae; genital papillae two-segmented, with rounded apices. Coxal setae (*4b*) situated at anterior tips of coxal apodemes IV; genital setae (*g*) laterad of genital opening. Attachment organ posterior to coxal fields IV. Anterior suckers (*ad*_3_) round, median suckers (*ad*_1+2_) distinctly larger, with paired vestigial alveoli (not situated on a common sclerite); pair of small refractile spots anterolateral to median suckers (*ps*_3_); lateral conoidal setae of attachment organ (*ps_2_*) situated slightly posterior to line joining centers of median suckers, distinctly anterior conoidal setae (*ps*_1_) and slightly posterior to median suckers (*ad*_1+2_); anterior and posterior lateral and posterior median cuticular conoids well-developed; anus situated between anterior suckers (*ad*_3_).

Legs. Legs elongate, all segments free. Trochanters I–III each with long, filiform seta, *pR* I–II, *sR* III. Femoral setation 1-1-0-1; setae *vF* I–II and *wF* IV long, filiform. Genual setation 2-2-0-0; setae *mG* and *cG* I–II filiform, seta *nG* III absent. Tibial setation 2-2-1-1; setae *hT* I and II spiniform; setae *gT* I filiform, long (reaching the base ω_3_ I); *gT* II shorter, filiform; setae *kT* III somewhat spiniform, *kT* IV and has a distinct prong. Tarsal setation 7-8-8-8. All pretarsi consisting of hooked empodial claws arising from tarsal apices, and short, paired condylophores within tarsal apices. Tarsus I with three filiform setae (*p*, *q*, and *d*), three slightly foliate (*ra*, *la*, and *f*), and one spoon-shaped seta *e*; seta *d* elongated, longer than tarsus (its base situated at level of bases *ra* and *la*); seta *s* alveolar; setae *wa*, *aa*, and *ba* I absent; tarsus II similar to tarsus I except seta *ba* present and filiform, base of seta *d* posterior of bases *ra* and *la*. Tarsus III with eight setae (*w*, *r*, *s*, *p*, *q*, *e*, *f*, *d*) smooth; all setae, except *d* III foliate. Tarsus IV similar to tarsus III, except seta *w* filiform, with a distinct prong. Solenidia ω_1_ on tarsi I–II cylindrical, with slightly clavate apices, ω_1_ II longer than ω_1_ I. Solenidion ω_3_ on tarsus I longer and thinner than ω_1_, with rounded apex, positioned slightly anterior to ω_1_; ω_1_ and ω_3_ separated by bulbous famulus (ε). Solenidion ω_2_ of tarsus I thin, slightly widened apically, situated somewhat more basal and posterior to ω_1_+ε+ ω_3_ group. Solenidia φ of tibiae I–III elongate, tapering; φ I and III longer than tarsus I and III, respectively; φ II shorter than tarsus II; φ IV short. Solenidia σ of genu I elongate, slightly tapering, nearly reaching tip of tibia I; σ of genu II shorter, cylindrical, not reaching midlength of tibia II; σ of genu III absent.

**Diagnosis**

Female. *Thyreophagus berxi* is close to *Th. spinitarsis* (Fain, 1982) and *Th. potawatomorum* sp. n. by tarsi III with seven spiniform setae (*e*, *u*, *v*, *p*, *q*, *s*, *w*) and the prodorsal sclerite with linear striation extending over at least 75% of its length. *Th. berxi* differs from *Th. spinitarsis* by the vase-shaped atrium of the spermatheca, with the width in the central part two-times shorter than the width at the entrance to spermatheca (dome-shaped, width in central part vs. basal part is subequal in *Th. spinitarsis*). See above for the differences from *Th. potawatomorum*.

Heteromorphic deutonymph. *Th. berxi* is close to *Th. corticalis* and *Th. potawatomorum* by the following characters: body elongate (more than 1.7-times longer than wide), setae *hT* and *gT* I–II present, different in shape, bases of seta *d* are at the level of bases of *ra* and *la* on tarsus I. *Th. berxi* differs from *Th. corticalis* by the following character states: the posterior medial apodeme is weakly developed (absent in *Th. corticalis*); the diameter of ocelli is about 23 (19 in *Th. corticalis*); setae *wa* I are absent (present in *Th. corticalis*); seta *kT* IV with a distinct prong (vs. prong absent in *Th. corticalis*). See above for the differences from *Th. potawatomorum*.

## 4. Keys to Species of *Thyreophagus* of the World

Females

Adults of the following species are unknown: *Th. africanus* (Mahunka, 1974), *Th. javensis* (Oudemans, 1911), *Th. sminthurus* (Fain and Johnston, 1974), *Th. johnstoni* (Fain, 1982), *Th. leclercqi* (Fain, 1982), *Th. rwandanus* (Fain, 1982).

Not included (*species inquirendae*): *Th. aleurophagus* (Sicher, 1894), *Th. angustus* (Banks, 1906), *Th. berlesianus* (Zachvatkin, 1941), *Th. entomophagus nominalis* (Kadzhaya, 1973), *Th. lignieri* (Zachvatkin, 1953), *Th. magnus* (Berlese, 1910), *Th. polezhaevi* (Zachvatkin, 1953), *Th. ponticus* (Kadzhaya, 1973).

1 Very large species, body length > 1500 μm. Egypt………………………………………………………………………………*Th. cynododactylon* (El-Bishlawy, 1990).

- Smaller species, body length < 700 μm………………………………………………………………………………………………………………………………………2

2 Setae *u* and *v* on tarsi III–IV vestigial; seta *wF* IV absent. USA (Florida) …………………………………………………………………………………*Th. hobe* sp. n.

- Setae *u* and *v* on tarsi III–IV well-developed, spiniform; seta *wF* IV present……….……………………………………………………………………………………3.

3 Tarsus III with three ventral apical spiniform setae (*s*, *u* and *v*) well-developed, proral setae *p* and *q* vestigial or absent…………………………………………4.

- Tarsus III with five ventral apical spiniform setae well-developed (*s*, *u*, *v*, *p*, and *q*)……………………………………………………………………………………7.

4 Prodorsal sclerite wider than long, almost entirely punctate, with a few short longitudinal striations in posteromedian region; atrium of spermatheca in form of an inverted bell, with base 18–20 μm wide; seta *w* III filiform. Widespread…………………………………………………………………………*Th. entomophagus* (Laboulbène and Robin, 1862).

- Prodorsal shield distinctly longer than wide, almost entirely covered with fine longitudinal striations; atrium of spermatheca smaller, not in form of a bell; seta *w* III small spine or absent…………………………………………………………………………………………………………………………………………………………………………………5

5 Prodorsal sclerite with linear striation in posterior half of shield. Italy……………………………………………………………………*Th. italicus* (Vacante, 1989).

- Prodorsal sclerite with linear striation extending over at least 75% of its length………………………………………………………………………………………6

6 Idiosomal length 270–360 μm, width 87–150 μm; atrium of spermatheca dome-shaped, wider (6 μm) than long (5 μm) and not narrowed toward its center; seta *w* III a very short spine; seta *se* not longer than prodorsal shield. Morocco………………………………………………………………………………………………………*Th. cooremani* (Fain, 1982).

- Idiosomal length 525–675 μm, width 210–280 μm; atrium of spermatheca vase-shaped, 12 μm long, maximum width 12 μm, narrowed toward the center where it 5 μm wide; narrowed toward middle and widened in its proximal part; seta *w* III vestigial; seta *se* longer than prodorsal sclerite. Europe…………………………..*Th. odyneri* (Fain, 1982).

7 Seta *w* III filiform…………………………………………………………………………………………………………………………………………………………………8

- Seta *w* III spiniform or vestigial…………………………………………………………………………………………………………………………………………………13

8 Anterior margin of prodorsal sclerite without paired indentations…………………………………………………………………………………………………………9

- Anterior margin of prodorsal sclerite with paired indentations……………………………………………………………………………………………………………10

9 Atrium of spermatheca weakly developed; sclerotized portion of spermatheca in form of a broad arc, much wider than long. Cuba …*Th. passerinus* (Cruz, 1990).

- Atrium of spermatheca well-developed, vase-shaped. Kenya…………………………………………………*Th. plocepasseri* (Klimov, Mwangi, Vangansbeke, 2020).

10 Seta *wa* I absent. Ukraine…………………………………………………………………………………………………………*Th. annae* (Sevastianov and Kivganov, 1992).

- Seta *wa* I present, spiniform or vestigial………………………………………………………………………………………………………………………………………11

11 Solenidion σ’ I longer than σ’’ I; prodorsal sclerite at most 1.3-times as long as wide...................................................................................................................12

- Solenidion σ’ I shorter than σ’’ I; prodorsal sclerite 1.5-times longer than wide. Ireland……………………………………………………………*Th. evansi* (Fain, 1982).

12 Long terminal setae *h*_2_ and *h*_3_ with bases inflated, conical; base of spermatheca forming thin sclerotized arc that divided anteriorly into four short, fine sclerotized lines; genu I with solenidion σ’ short, 8 μm long, σ” 6 μm long (ratio 1.4:1); Great Britain ……………………………………………………………………………*Th. macfarlanei* (Fain, 1982).

- Long terminal setae *h*_2_ and *h*_3_ with very thin bases; atrium U-shaped with thick sides, 6 μm long, 5 μm wide; genu I with solenidion σ’ thin, 18–20 μm, σ” slightly thickened, 12 μm long (ratio 1.58: 1); Morocco …………………………………………………………………………………………………………………………………*Th. athiasae* (Fain, 1982).

13 Seta *w* III well-developed, spiniform…………………………………………………………………………………………………………………………………………14

- Seta *w* III vestigial………………………………………………………………………………………………………………………………………………………………25

14 Solenidion σ II very short, 2–3-times longer than its width; atrium absent or minute, much shorter than sclerites of oviducts (in *Th. ais*). Base of spermatheca in form of a broad arc, much wider than long, or small and rounded …………………………………………………………………………………………………………………………………………15

- Solenidion σ II longer; atrium, well-developed, longer than wide. Base of spermatheca variable …………………………………………………………………19

15 Solenidion σ II short and filiform or nearly conical, but with sides straight and not convex………………………………………………………………………16

- Solenidion σ II with convex sides…………………………………………………………………………………………………………………………………………17

16 Solenidion σ II short and filiform or nearly conical, but with sides straight and not convex; linear sclerites near the typical sclerites of oviducts present; paired sclerites of oviducts Y-shaped, not elongated, at least three-times longer than its width. Widespread ………………………………………………………...*Th. gallegoi* (Portus and Gomez, 1979).

- Solenidion σ II short and filiform; linear sclerites near the typical sclerites of oviducts absent; paired sclerites of oviducts elongated, more than five-times longer than their width, V-shaped. Great Britain ……………………………………………………………………………………………………………………………………*Th. vermicularis* (Fain and Lukoschus, 1982).

17 Paired sclerites of oviducts V-shaped; canal of spermatheca at entrance to spermatheca widened; bases of setae *vi* nearly touching, situated in common unsclerotized area. USA (Florida)…………………………………………………………………………………*Th. Calusorum* (Klimov, Demard, Stinson, Duarte, Wäckers, and Vangansbeke, 2022).

- Paired sclerites of oviducts Y-shaped; canal of spermatheca at entrance to spermatheca, uniform in width, not widened; bases of setae *vi*, separated, not in common area …………………………………………………………………………………………………………………………………………………………………………………………18

18 Cuplike portions of sclerites of oviducts distinctly shorter than their stems; solenidia σ’ and σ’’ subequal; solenidion ω_1_ II five-times longer than its width; solenidion φ IV reaching middle of tarsus IV. Mauritius ………………………………………………………………………………………………………………………………*Th. mauritianus* (Fain, 1982).

- Cuplike portions of sclerites of oviducts subequal; solenidia σ’ distinctly longer than σ’’; solenidion ω_1_ II three-times longer than its width; solenidion φ IV longer, nearly reaching bases of setae *d* IV. USA (Florida) ……………………………………………………………………………………………………………………………………………*Th. ais* sp. n.

19 Seta *wa* I present…………………………………………………………………………………………………………………………………………………………………20

- Seta *wa* I absent…………………………………………………………………………………………………………………………………………………………………………21

20 Anterior margin of prodorsal shield without paired indentations; prodorsal shield smoothly punctated; posterior hysterosomal seta *h*_1_ less than half of length of *h*_2_. Colombia………………………………………………………………………………………………………………………………………*Th. incanus* (Fain and Rack, 1987).

- Anterior margin of prodorsal shield with paired indentations; prodorsal shield with linear striation extending over at least 75% of its length; posterior hysterosomal seta *h*_1_ half of length of *h*_2_. Europe……………………………………………………………………………………………………………………………………………………*Th. spinitarsis* (Fain, 1982).

21 With one pair of large, sclerotized, funnel-like, internal structures near posterior end of body (not to be confused with sclerites oviducts) ………………………22

- Without paired, funnel-like structures in posterior body……………………………………………………………………………………………………………………………23

22 Solenidion φ of tibia IV very short (4 μm); USA (California) …………………………………………………………………………*Th. tridens* (Fain and Lukoschus, 1986).

- Solenidion φ of tibia IV longer (14 μm). Brazil…………………………………………………………………………*Th. cracentiseta* Barbosa, (OConnor and Moraes, 2016).

23 Setae *h*_1_, *h*_2_, and *h*_3_ very long (2.5–2.8-times longer than length of prodorsal shield), similar in length. New Zealand……………………………*Th. australis* (Clark, 2009).

- Setae *h*_2_ and *h*_3_ less than 2.5-times longer than length of prodorsal shield, seta *h*_1_ less than half of length of *h*_2_………………………………………………………………24

24 Setae *vi* short, not extending beyond anterior margin of prodorsum; bases of *vi* touching; medial area of prodorsal shield with non-uniform pattern of striations in its anterior and posterior parts; setae *h*_2_ and *h*_3_ shorter than anus; rounded sclerotized capsule at junction of canal of spermatheca and atrium present. USA (Michigan)………………………………………………………………………………………………………………………………………………………*Th. potawatomorum* sp. n.

- Setae *vi* long, extending beyond anterior margin of prodorsum; bases of setae *vi* distinctly separated; entire medial area of prodorsal shield uniformly striated; setae *h*_2_ and *h*_3_ longer than anus; rounded capsule at junction of canal of spermatheca and atrium absent. France, Belgium, Germany…*…………………………………………………………Th. berxi* sp. n.

25 Setae *h*_2_ and *h*_3_ 1.1–1.25-times longer than length of prodorsal shield; diameter of genital papillae approximately six-times shorter than length of genital setae. Palaearctic ………………………………………………………………………………………………………………………………………………………………………*Th. corticalis* (Michael, 1885).

- Setae *h*_2_ and *h*_3_, long, their lengths 1.75-times longer than length of prodorsal shield; diameter of genital papillae approximately four-times shorter than length of genital setae. USA (Michigan), Canada (Ontario) …*………………………………………………………………………………………………………………………………………Th. ojibwe* sp. n.

Males (modified after [1])

Adults of the following species are unknown: *Th. africanus* (Mahunka, 1974), *Th. javensis* (Oudemans, 1911), *Th. sminthurus* (Fain and Johnston, 1974), *Th. johnstoni* (Fain, 1982), *Th. leclercqi* (Fain, 1982), and *Th. rwandanus* (Fain, 1982).

Not included (*species inquirendae*): *Th. aleurophagus* (Sicher, 1894), *Th. angustus* (Banks, 1906), *Th. berlesianus* (Zachvatkin, 1941), *Th. entomophagus nominalis* (Kadzhaya, 1973), *Th. lignieri* (Zachvatkin, 1953), *Th. magnus* (Berlese, 1910), *Th. polezhaevi* (Zachvatkin, 1953), and *Th. ponticus* (Kadzhaya, 1973).

Males are unknown in *Th. athiasae*, *Th. plocepasseri*, *Th. polezhaevi*, *Th. cooremani*, Th. *calusorum*, *Th. evansi*, *Th. macfarlanei*, *Th. spinitarsis*, *Th. tridens*, *Th. vermicularis*, *Th. ais*, *Th. berxi*, *Th. hobe*, *Th. potawatomorum*, and *Th. mauritianus*.

Not included (*species inquirendae*): *Th. aleurophagus*, *Th. angustus*, *Th. berlesianus*, *Th. italicus*, *Th. lignieri*, *Th. magnus*, and *Th. ponticus*.

1 Prodorsal sclerite smoothly punctulate, without longitudinal striations……………………………………………………………………………………………………………2

- Prodorsal sclerite with short longitudinal striations, at least near posterior margin…………………………………………………………………………………………………5

2 Posterior venter with sclerotized projection very poorly developed or absent. Colombia…………………………………………………….*Th. incanus* (Fain and Rack, 1987).

- Posterior venter with sclerotized projection well-developed…………………………………………………………………………………………………………………………3

3 Body elongate, six-times longer than wide; large species, length > 700 μm. Egypt……………………………………………….*Th. cynododactylon* (El-Bishlawy, 1990).

- Body ovoid, 1.5–2-times longer than wide; small species, length < 500 μm. Widespread……………………………………………………………………………………………...4

4 Tarsus IV with five spine-like setae (*s*, *u*, *v*, *p*, *q*), three filiform setae (*f*, *r*, *w*), and two suckers (*d*, *e*). Ireland…………………………………………………….*Th. evansi* (Fain, 1982).

- Tarsus IV with three spine-like setae (*s*, *u*, *v*), three filiform setae (*f*, *r*, *w*), and two suckers (*d*, *e*). Widespread………………*Th. entomophagus* (Laboulbène and Robin, 1862).

5 Posterior venter with distinct rounded, sclerotized projection…………………………………………………………………………………………………………………………6

- Posterior body smoothly rounded, without ventral projection…………………………………………………………………………………………………………………………8

6 Entire width of prodorsal sclerite covered by longitudinal striation. Europe……………………………………………………………………………*Th. odyneri* (Fain, 1982).

- Longitudinal striation on prodorsal sclerite restricted to median region, lateral areas simply punctulate……………………………………………………………………………7

7 Setae *h*_2_ and *h*_3_ 1.1–1.25-times longer than length of prodorsal shield; spines *p*, *q*, and *u*, *v* III–IV subequal. Palaearctic…………………………..*Th. corticalis* (Michael, 1885).

- Setae *h*_2_ and *h*_3_ long, their lengths are 1.8-times longer than the length of the prodorsal shield; spines *p* and *q* shorter than spines *u* and *v* on tarsi III–IV. USA (Michigan), Canada (Ontario). …………………………………………………………………………………………………………………………………………………………………………………*Th. ojibwe* sp. n.

8 Posterior hysterosoma with a large sclerotized area extending posteriad from level of setae *e*_2_. Ukraine……………………………..*Th. annae* (Sevastyanov and Kivganov, 1992).

- Posterior hysterosoma unsclerotized or at most with short terminal sclerotization posterior to setae *h*_1_…………………………………………………………………………9

9 Posterior idiosoma with short sclerotized area posterior to setae *h*_1_…………………………………………………………………………………………………………………10

- Posterior idiosoma unsclerotized…………………………………………………………………………………………………………………………………………………………11

10 Genu I with solenidia σ’ and σ” approximately equal in length. Widespread……………………………………………………………*Th. gallegoi* (Portus and Gomez, 1979).

- Genu I with solenidion σ’ only half of length of σ”. Cuba………………………………………………………………………………………………*Th. passerinus* (Cruz, 1990).

11 Dorsal hysterosomal setae relatively long, setae *d*_2_ and *e*_2_ much longer than distance between their alveoli. New Zealand………………………*Th. australis* (Clark, 2009).

- Dorsal hysterosomal setae much shorter, setae *d*_2_ and *e*_2_ shorter than distance between their alveoli. Brazil…*…….Th. cracentiseta* (Barbosa, Oconnor, and Moraes, 2016).

Heteromorphic deutonymphs

Unknown for the following species: *Th. aleurophagus*, *Th. angustus*, *Th. annae*, *Th. athiasae*, *Th. cooremani*, *Th. cracentiseta*, *Th. cynododactylon*, *Th. entomophagus nominalis*, *Th. evansi*, *Th. gallegoi*, *Th. hobe*, *Th. incanus*, *Th. italicus*, *Th. macfarlanei*, *Th. magnus*, *Th. mauritianus*, *Th. ojibwe*, *Th. odyneri*, *Th. passerinus*, *Th. plocepasseri*, *Th. polezhaevi*, *Th. ponticus*, *Th. spinitarsis*, *Th. tridens*, and *Th. vermicularis*.

Not included (*species inquirendae*): *Th. berlesianus*, *Th. lignieri*.

Published measurements of *T. corticalis* (distance between ocelli) were corrected.

1 Dorsal surface completely striated. Afrotropical………………………………………………………………………………………………………………*Th. africanus* (Mahunka, 1974).

- Dorsal surface smoothly punctate, without striations…………………………………………………………………………………………………………………………………………2

2 Body ovoid, 1.3–1.5-times longer than wide…………………………………………………………………………………………………………………………………………………3

- Body elongate, more than 1.7-times longer than wide…………………………………………………………………………………………………………………………………………5

3 Seta *hT* I more than half the length of *gT* I. Europe………………………………………………………………………………………………………………….*Th. leclercqi* (Fain, 1982).

- Seta *hT* I less than half the length of *gT* I……………………………………………………………………………………………………………………………………………………4

4 Opisthonotal gland openings approximately equidistant from setae *c*_3_ and *c*_p_; seta *kT* III filiform; setae *wa* I–II absent. Widespread…*Th. entomophagus* (Laboulbène and Robin, 1862).

- Opisthonotal gland openings much closer to ventral seta *c*_3_ than to dorsolateral seta *c*_p_; seta *kT* III with distinct prong; setae *wa* I-II present. New Zealand…*Th. australis* (Clark, 2009).

5 Hysterosomal sclerite about 1.7-times longer than prodorsal sclerite…………………………………………………………………………………………………………………6

- Hysterosomal sclerite about two-times longer than prodorsal sclerite…………………………………………………………………………………………………………………7

6 Seta *hT* I absent; posterior medial apodeme in area of coxal fields IV present; seta *kT* III with distinct prong. Great Britain, USA (Washington) …*Th. sminthurus* (Fain and Johnston, 1974).

- Seta *hT* I present; posterior medial apodeme in area of coxal fields IV absent; seta *kT* III without prong, filiform. USA (Maryland)………………………*Th. johnstoni* (Fain, 1982).

7 Setae *hT* II and *gT* II subequal ………………………………………………………………………………………………………………………………………………………………………8

- Setae *hT* II twice the length of *gT* II…………………………………………………………………………………………………………………………………………………………………9

8 Setae *hT* II and *gT* II filiform, both subequal to tibia II. Java………………………………………………………………………………………………*Th. javensis* (Oudemans, 1911).

- Setae *hT* II and *gT* II spiniform, shorter than half the length of tibia. Afrotropical………………………………………………………………………………*Th. rwandanus* (Fain, 1982).

9 On tarsus I, bases of setae *d* situated at the same level with bases of setae *ra* and *la*; diameter of ocellus 16–23 μm …………………………………………………………………10

- On tarsus I, bases of setae *d* distal to bases of setae *ra* and *la*; diameter of ocellus 10–14 μm …………………………………………………………………………………………….12

10 Width of ocellus 16 μm, distance between ocelli 36 μm; posterior medial apodeme in area of coxal fields IV present. USA (Michigan)………………..*Th. potawatomorum* sp. n.

- Width of ocellus about 18–23 μm, distance between ocelli about 40–50 μm; posterior medial apodeme in area of coxal fields IV weakly developed or absent………………….11

11 On tibia IV, seta *kT* IV with distinct prong; width of ocellus 23 μm, distance between ocelli 50 μm; posterior medial apodeme in area of coxal fields IV weakly developed. Europe……………………………………………………………………………………………………………………………………………………………………………………*Th. berxi* sp. n.

- On tibia IV, seta *kT* IV without prong; width of ocellus about 18–19 μm, distance between ocelli about 40–42 μm; posterior medial apodeme in area of coxal fields IV absent. Widespread………………………………………………………………………………………………………………………………………………………………*Th. corticalis* (Michael, 1885).

12 Tarsal setae *p* and *q* on tarsus IV foliate; seta *d* III shorter than leg III; setae *d* IV shorter or slightly longer than leg IV; diameter of ocellus 10–13. USA (Florida)……………………………………………………………………………………………………………………*Th. calusorum* (Klimov, Demard, Stinson, Duarte, Wäckers and Vangansbeke, 2022)

- Tarsal setae *p* and *q* on tarsus IV short, spiniform; setae *d* III longer than leg III, *d* IV distinctly longer than leg IV; diameter of ocellus 12–14. USA (Florida)…*…….Th. ais* sp. n.

## Data Availability

No new data were created or analyzed in this study. Data sharing is not applicable to this article.
